# Convergent NMDA receptor—Pannexin1 signaling pathways regulate the interaction of CaMKII with Connexin-36

**DOI:** 10.1038/s42003-021-02230-x

**Published:** 2021-06-08

**Authors:** Ryan C. F. Siu, Anna Kotova, Ksenia Timonina, Christiane Zoidl, Georg R. Zoidl

**Affiliations:** 1 Department of Biology, York University, Toronto, ON Canada; 2 Center of Vision Research, York University, Toronto, ON Canada; 3 Department of Psychology, York University, Toronto, ON Canada

**Keywords:** Molecular neuroscience, Cellular neuroscience

## Abstract

Ca^2+^/calmodulin-dependent protein kinase II (CaMKII) binding and phosphorylation of mammalian connexin-36 (Cx36) potentiate electrical coupling. To explain the molecular mechanism of how Cx36 modifies plasticity at gap junctions, we investigated the roles of ionotropic N-methyl-D-aspartate receptors and pannexin1 (Panx1) channels in regulating Cx36 binding to CaMKII. Pharmacological interference and site-directed mutagenesis of protein interaction sites shows that NMDA receptor activation opens Cx36 channels, causing the Cx36- CaMKII binding complex to adopt a compact conformation. Ectopic Panx1 expression in a Panx1 knock-down cell line is required to restore CaMKII mediated opening of Cx36. Furthermore, blocking of Src-family kinase activation of Panx1 is sufficient to prevent the opening of Cx36 channels. Our research demonstrates that the efficacy of Cx36 channels requires convergent calcium-dependent signaling processes in which activation of ionotropic N-methyl-D-aspartate receptor, Src-family kinase, and Pannexin1 open Cx36. Our results add to the best of our knowledge a new twist to mounting evidence for molecular communication between these core components of electrical and chemical synapses.

## Abstract (web summary)

Siu et al use FRET imaging in neuronal cell lines to explore the interplay between the gap junction channel protein Connexin-36 (Cx36) and NMDA receptor and Pannexin 1-mediated calcium signaling. They demonstrate that an increase in intracellular calcium promotes binding of CaMKII to Cx36, leading to increased gap junction opening, thus providing a pathway by which components of chemical and electrical synapses communicate.

## Introduction

Electrical synapses in neurons coexist alongside chemical synapses in mixed synapses throughout the vertebrate nervous system^1,2^. In the mammalian brain, most electrical synapses are composed of connexin-36 (Cx36) gap junction channels (GJC)^3^. Cx36 GJCs show functional plasticity similar to chemical synapses^4,5^, in which the interaction of Cx36 with Ca^2+^-activated calmodulin (CaM) and calmodulin protein kinase II (CaMKII) is considered analogous to the interaction of ionotropic N-methyl-d-aspartate (NMDA) receptors with CaM/CaMKII^6^.

CaMKII binding and phosphorylation of Cx36 have been identified as drivers of adaptive electrical coupling in neurons of teleosts and rodents. The protein motifs found in the cytoplasmic and carboxyterminal domains of Cx36 share a remarkable similarity to segments of the regulatory subunit of CaMKII^6,7^. Similar to the NR2B subunit of the NMDA receptor, both Cx36 binding sites exhibit phosphorylation-dependent interaction and autonomous activation of CaMKII, suggesting that the functional efficacy of both modes of interneuronal communication share common molecular features. Here, we explored the idea that Cx36 and NMDA receptor activities could be synchronized by sharing molecular features. Specifically, we hypothesized that ionotropic NMDA receptors mediate both synaptic potentiation and Cx36 activity by the timing of neuronal activity when glutamate release and a post-synaptic depolarization coincide temporally with calcium influx. The ATP-release channel Pannexin1 (Panx1) was tested as a potential mediator between NMDA receptors and Cx36. Panx1 is localized in glutamatergic synapses^8,9^ in the proximity of NMDA receptors and has been linked to the propagation of calcium waves^10^. Panx1, together with metabotropic and ionotropic NMDA receptors and Src family kinases (SFKs) have been implicated in forming signaling complexes^11–13^.

Here, we describe the identification of a signaling pathway in which activation of ionotropic NMDA receptors modulate the interaction between Cx36 and CaMKII. Individual steps in the signaling process include activation of the ATP-release channel Panx1^14–16^, SFKs^17^, and elevated intracellular calcium ([Ca^2+^]_I)_. Förster resonance energy transfer (FRET) was used to identify steps at which signaling converges onto the Cx36–CaMKII interaction complex. This technology allowed to determine changes to the compact conformation of the three-dimensional gap junction plaque (GJP) superstructure at an nm-resolution when the interaction between the dodecameric Cx36 GJC and the dodecameric CaMKII holoenzyme was tuned to activation or deactivation. Functional dye transfer assays and ethidium bromide fluorescence recovery after photobleaching^18^ were used to correlate functional changes with structural rearrangements in the protein complexes.

Results emphasize that the ionotropic NMDA receptor-mediated rise in [Ca^2+^]_I_ was an essential signal requirement to increase a compact conformation of the Cx36–CaMKII complex and to open Cx36 channels. Mutations in protein domains targeting the interaction between Cx36 and either CaM or CaMKII, and pharmacological interventions disrupting the NMDA receptor or CaMKII activation, abolished [Ca^2+^]_I_ signaling the opening of Cx36 channels located in GJPs. Inhibition of SFK, main actors in pathways leading to the generation of Ca^2+^ signals, and modulators of NMDA receptor^17^, the CaMKII holoenzyme^19^, and Panx1^20^, blocked Cx36 channel opening in this study. The genetic ablation of Panx1 was effective in closing Cx36 as was the blockage of NMDA receptors, SFK, or CaMKII, an effect that was rescued by overexpression of Panx1. We conclude that ionotropic NMDA receptors and Panx1 provide a signaling mechanism endowing mixed chemical and electrical synapses with the flexibility to control synaptic outputs.

## Results

### Calcium modulates proximity among Cx36 channels in GJPs

**Fig. 1:**
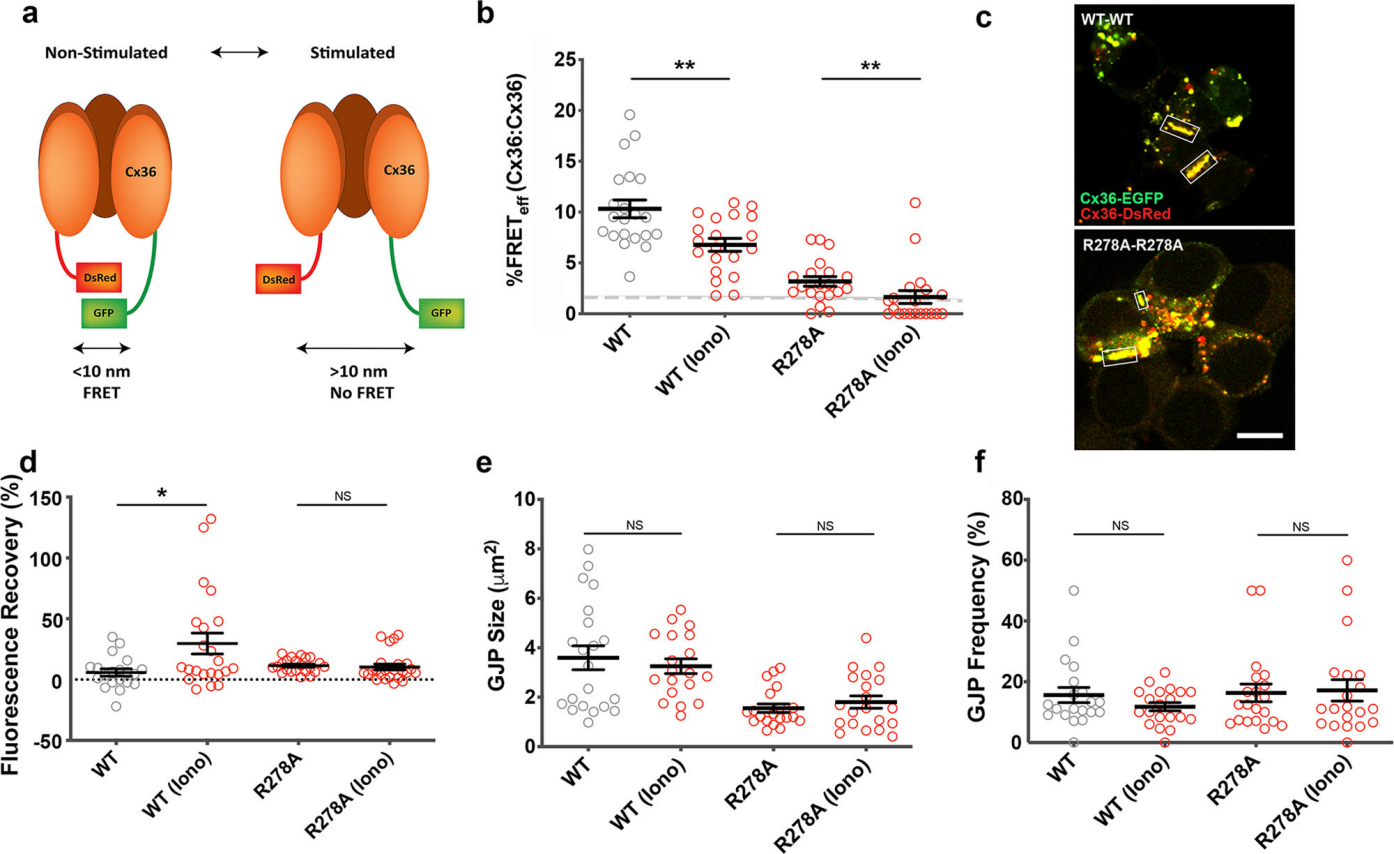
Calcium modulates proximity between Cx36 channels when CaM/CaMKII binding is intact. **a** Anticipated changes to the compactness of Cx36 channels located in GJPs when the distance between FRET pairs increases after channels open. **b** FRET_eff_ of wild-type (WT) or the mutant R278A with and without treatment with 2 µM ionomycin and 2 mM extracellular calcium. The dashed line indicates the 1.7% or 10 nm limit of FRET. **c** Expression and localization of Cx36–DsRed/Cx36–EGFP and Cx36–DsRed/Cx36 R278A in double transfected Neuro2a cells. GJPs are highlighted with rectangles. Scale bar: 10 µm. **d** Functional assessment of Cx36 channel opening by photobleach recovery of cytoplasmic ethidium bromide. **e**, **f** Quantification of gap junction plaque size and frequency. Beeswarm graphs show mean ± SEM; Mann–Whitney *U* (two-tailed) significance test, **p < 0.01, *p < 0.05. NS not significant, Iono ionomycin.

Opening and closing of Cx36 channels were expected to affect the space occupied in the three-dimensional crystal-like structure of GJPs (Fig. 1a). FRET was used to compare the short-range structural changes between wildtype Cx36 with the CaM/CaMKII binding deficient mutant, Cx36 R278A^18^, in response to increased [Ca^2+^]_I_. At normal cell growth conditions, Cx36–EGFP and Cx36–DsRed wildtype or Cx36–EGFP R278A and Cx36–DsRed R278A pair fluorescence was found in GJPs at comparable amounts (Supplementary Fig. S1). The FRET efficiency (FRET_eff_) of Cx36–EGFP and Cx36–DsRed pairs were significant, suggesting the proximity of the fluorescent protein tags when Cx36 channels were packed in GJPs. Elevation of [Ca^2+^]_I_ by treatment with 2 µM ionomycin in the presence of 2 mM extracellular calcium [Ca^2+^]_E_ decreased FRET_eff_, consistent with an increase in distance between the fluorescent protein tags corresponding to the opening of Cx36 GJC (non-stimulated: 10.3 ± 0.9, *n* = 21; ionomycin: 6.8 ± 0.6, *n* = 20, *p* = 0.007) (Fig. 1b, c: upper image). Co-expressed CaM/CaMKII binding deficient mutants Cx36–EGFP R278A and Cx36–DsRed R278A had low FRET_eff_ regardless of Ca^2+^ stimulation (non-stimulated: 3.2 ± 0.5, *n* = 20; ionomycin: 1.6 ± 0.6, *n* = 20, *p* = 0.003) (Fig. 1b, c: lower image).

Next, we tested the functional coupling of wild-type Cx36 and the R278A mutant using an ethidium bromide uptake and recovery after photobleaching assay^18,21^. Neuroblastoma 2a (Neuro2a) cell pairs expressing Cx36–EGFP or Cx36–EGFP R278A GJPs were selected to quantify the recovery of ethidium bromide fluorescence in the cytoplasm in 0.5–1 µm distance to GJPs after photobleaching one side of the channels (Supplementary Fig. S2). Neuro2a cells treated with ionomycin showed significant ethidium bromide fluorescence recovery in this region of interest when they expressed Cx36–EGFP (Fig. 1d) (non-stimulated Cx36–EGFP: 5.8 ± 3.0, *n* = 20; ionomycin: 29.6 ± 8.6, *n* = 22, *p* = 0.04; non-stimulated Cx36 R278A: 11.5 ± 1.2, *n* = 22; ionomycin: 10.3 ± 2.4, *n* = 24, *p* = 0.08).

Potential confounding factors, such as changes to GJP size and frequency caused by modulation of [Ca^2+^]_I_ were excluded. Both GJP size (Fig. 1e, f) (wild-type non-stimulated: 3.6 ± 0.5, *n* = 21; ionomycin: 3.3 ± 0.3, *n* = 19, *p* = 0.90) and GJP frequency (wild-type non-stimulated: 15.6 ± 2.5, *n* = 20; ionomycin: 11.8 ± 1.3, *n* = 20, *p* = 0.74) showed no significant difference with elevated [Ca]_I_. Cx36–EGFP R278A and Cx36–DsRed R278A mutant channels also formed GJPs and the frequency were unaffected by [Ca^2+^]_I_ stimulation (GJP size: R278A: non-stimulated: 1.6 ± 0.2, *n* = 20; ionomycin: 1.8 ± 0.2, *n* = 20, *p* = 0.75; GJP frequency: R278A: non-stimulated: 16.3 ± 2.9, *n* = 20; ionomycin: 17.2 ± 3.5, *n* = 20, *p* = 0.75). The GJP size of mutants was significantly smaller than wild-type Cx36, suggesting that loss of CaM/CaMKII binding affected Cx36 recruitment to GJPs_._ The results demonstrated proximity changes between Cx36 channels caused by increased [Ca^2+^]_I_, which require the CaM/CaMKII binding motif to converge calcium signals onto Cx36 GJCs.

### Calcium signals converge onto Cx36 through interaction with CaM/CaMKII

**Fig. 2:**
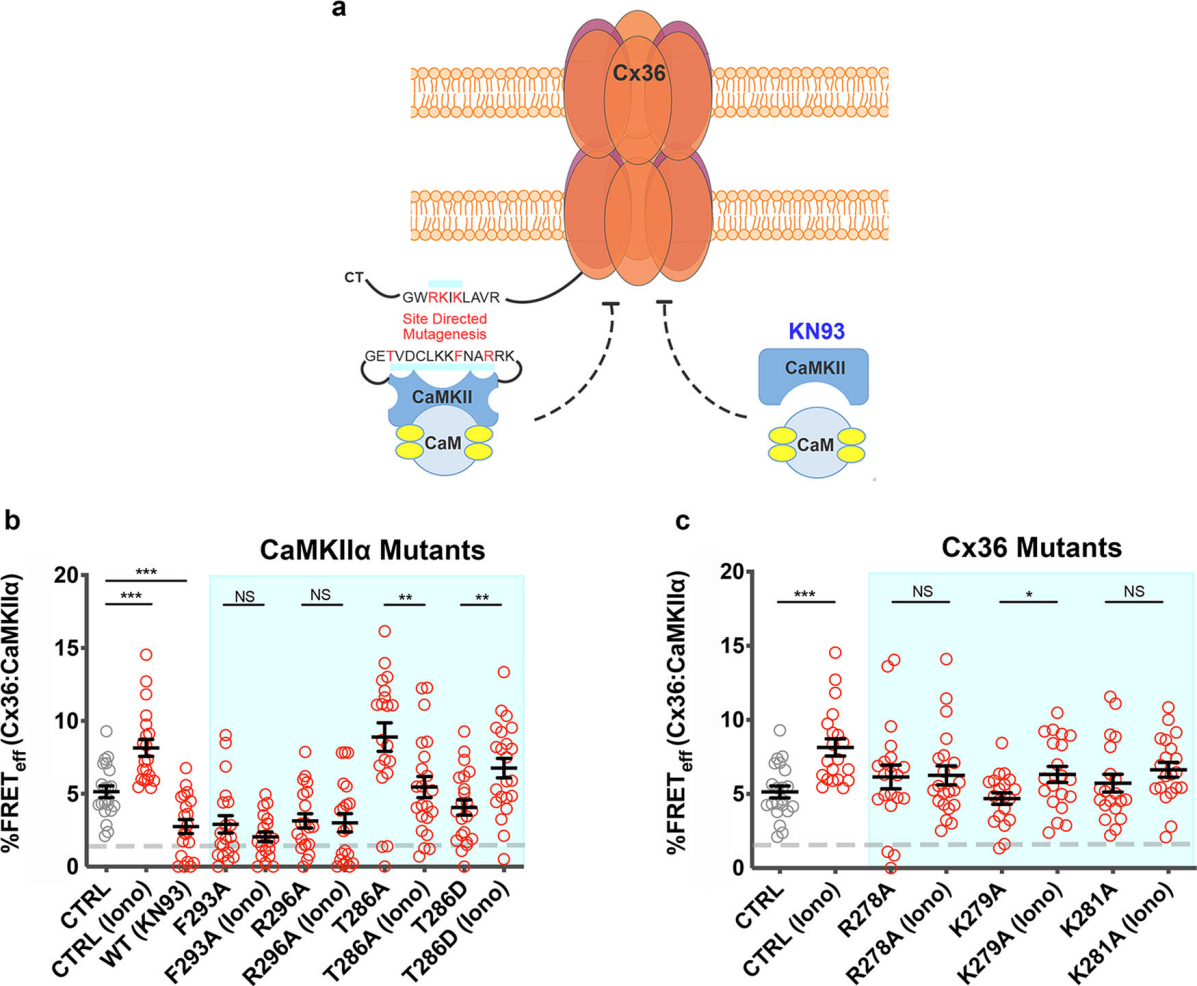
Cx36–CaMKII interaction at GJPs. **a** Visual summary of the interactions targeted pharmacologically (KN93) or genetically (site-directed mutagenesis) (blunt arrow = blockage/inhibition). **b** FRET_eff_ of Cx36–DsRed and WT or mutant CaMKIIα–ECFP in response to Ca^2+^ and pharmacological blocking of CaMKII with KN-93. **c** FRET_eff_ of WT and mutant Cx36–DsRed and WT and CaMKIIα–ECFP in response to Ca^2+^. Treatments in **b**, **c**: Iono (2 mM [Ca^2+^]_E_ and 2 μM ionomycin), KN93 (50 μM). Beeswarm graphs with mean ± SEM; Mann–Whitney *U* significance (two-tailed), ***p < 0.001, **p < 0.01, *p < 0.05, NS not significant.

Changes in the proximity between Cx36 and CaMKIIα in response to experimentally manipulated [Ca^2+^]_I_ were investigated by FRET. Cx36 and CaMKIIα share a conserved CaM binding region found across CaMKII isoforms (Supplementary Fig. S3a). Ca^2+^ effects on proximity of Cx36 to wild-type and mutant CaMKII were used to investigate the step at which Ca^2+^ activated CaM binds CaMKIIα (Fig. 2a). Elevation of [Ca^2+^]_I_ with 2 µM ionomycin caused significant FRET_eff_ responses (CTRL: 5.1 ± 0.4, *n* = 21; ionomycin: 8.1% ± 0.5, *n* = 20, *p* = 2.13 × 10^−4^) for Cx36 binding to CaMKIIα. Point mutations in the CaMKII regulatory region which alter the CaM footprint did not respond to treatment (F293A; non-stimulated: 2.9 ± 0.6, *n* = 20; stimulated: 2.0 ± 0.3, *n* = 20, *p* = 0.52; R296A; non-stimulated: 3.1 ± 0.5, *n* = 20; stimulated: 3.0 ± 0.6, *n* = 20, *p* = 0.51) (Fig. 2b). The increase in FRET_eff_ when CaM binds to CaMKII to form the calcium-activated CaM/CaMKII holoenzyme complex suggested a tighter binding to Cx36.

Cx36, similar to NMDA receptors, can bind the calcium-activated CaM/CaMKII complex, and autophosphorylation at amino acid T286 is required for orchestrating the binding of CaMKIIα to Cx36^6^. Pharmacological intervention with the CaMKIIα inhibitor KN93 decreased FRET_eff_ (non-stimulated: 5.1 ± 0.4, *n* = 21; KN93: 2.8 ± 0.5, *n* = 20, *p* = 0.001) (Fig. 2b), likely through changes in the CaMKIIα structure, relaxing the binding complex, or by blocking the CaM/CaMKII complex^22^. Interestingly, FRET_eff_ of the autophosphorylation negative CaMKIIα mutant T286A was much higher than controls. This suggested that the mutation locked the interaction between Cx36 and CaMKII in a compact conformation and that this step did not require autophosphorylation. When [Ca^2+^]_I_ increased, FRET_eff_ decreased, most likely indicating that Cx36 detached from the complex (non-stimulated: 8.9 ± 1.0, *n* = 20; ionomycin: 5.5 ± 0.7, *n* = 22, *p* = 0.01) (Fig. 2b). The CaMKIIα T286D mutant and the wild-type protein showed a similar but slightly dampened response to [Ca^2+^]_I_ increase (non-stimulated: 4.1 ± 0.5, *n* = 22; ionomycin: 6.8 ± 0.7, *n* = 22, *p* = 0.004), which was consistent with reports indicating that nearly full activation of this mutant can be achieved by minimal further stimulation by Ca^2+^/CaM or when the binding protein mutes the mutant^23^ (Fig. 2b). We concluded that Ca^2+^-induced structural changes caused by the binding of Cx36 to CaMKII require CaMKIIα autophosphorylation at T286.

Cx36 point mutations at R278 and K281 disrupted binding to CaMKIIα (Fig. 2c). We found no change in FRET_eff_ of either mutant with CaMKII when [Ca^2+^]_I_ was increased (R278A, non-stimulated: 6.1 ± 0.8, *n* = 20; ionomycin: 6.3 ± 0.6, *n* = 21, *p* = 0.95; K281A, non-stimulated: 5.7 ± 0.6, *n* = 20; ionomycin: 6.6 ± 0.5, *n* = 20, *p* = 0.09). GJP frequency and size showed no significant differences when mutants Cx36–R278A and Cx36–K281A were co-expressed with CaMKII (Supplementary Fig. S3c–e). The mutant Cx36–K279A showed a reduction in GJP frequency consistent with a previous report^24^.

### Calcium is required for the interaction of Cx36 with CaMKIIα at the GJP

**Fig. 3:**
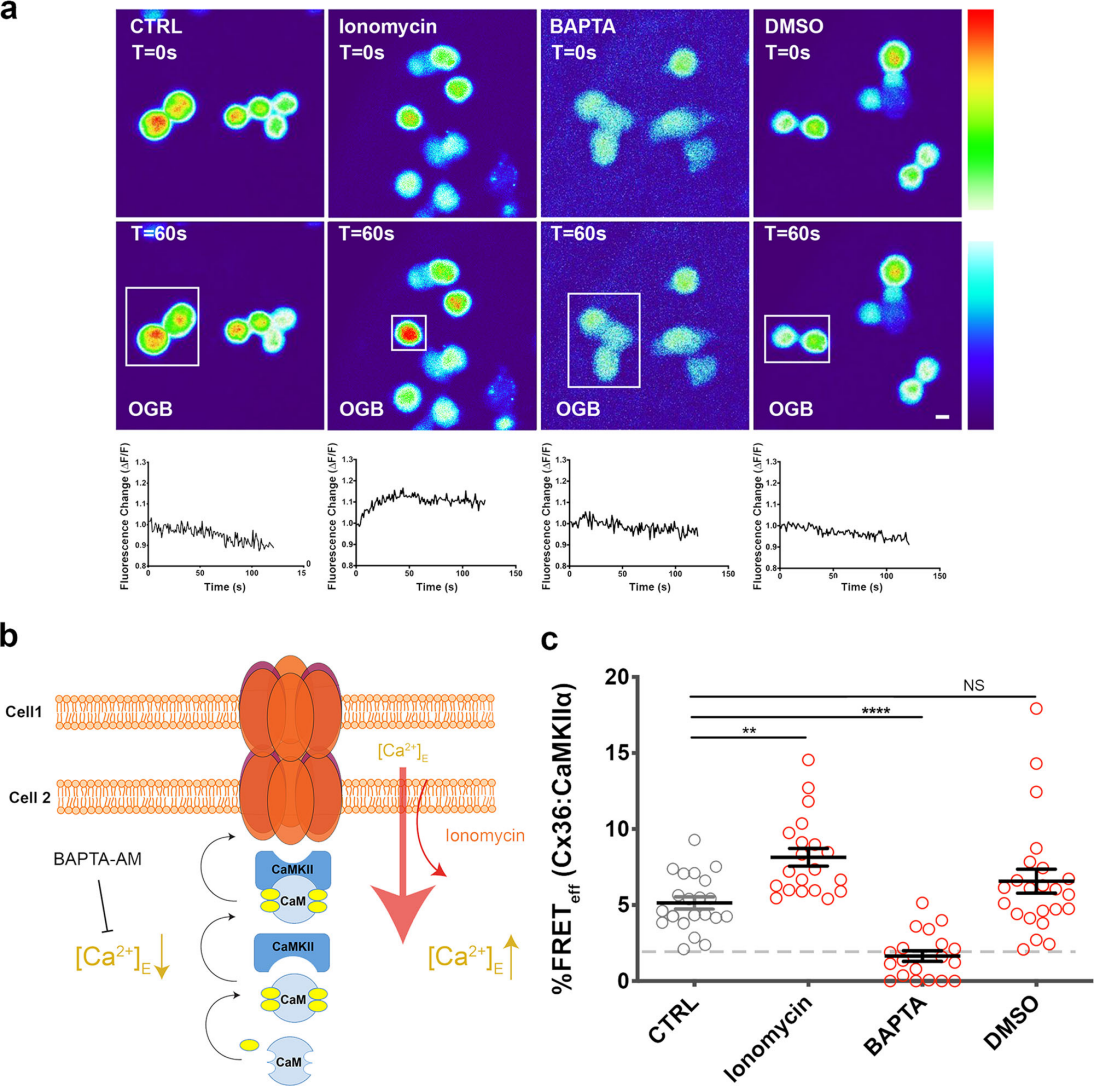
Calcium manipulation influences Cx36–CaMKIIα interaction. **a** Neuro2a cells labeled with Oregon green BAPTA-AM (OGB) showing calcium influx in response to pharmacological stimulants used in (**b**). The bottom row shows sample traces of relative fluorescence (Δ*F*/*F*) for ~120 s of selected cells (indicated by boxes) showing responses to treatments. Scale bar = 10 µm. **b** Neuro2a cells were co-transfected with Cx36–DsRed and CaMKIIα–ECFP and FRET efficiency (FRET_eff_) was determined 48 h post-transfection. The interaction between Cx36 and CaMKIIα was impacted by manipulating intracellular calcium levels (sharp arrows = direction of processes; blunt arrow = blockage/inhibition). **c** FRET_eff_ of Cx36:CaMKII after treatment with drugs added for 5 min (ionomycin) or 10 min (BAPTA) or 10 min DMSO as a negative control. Beeswarm graphs with mean ± SEM; dashed line indicates the 1.7% limit of FRET; Mann–Whitney *U* significance (two-tailed), ****p < 0.0001, **p < 0.01, NS not significant.

The fluorescent calcium reporter Oregon Green BAPTA-AM (OGB) was used next to visualize changes to [Ca^2+^]_I_ levels after treatment. Sample traces of treated Neuro2a cells, reporting fluorescence changes over 120 s, confirmed ionomycin-induced Ca^2+^ influx (boxed to show the most substantial changes). DMSO or BAPTA treatments caused negligible differences compared to the control (Fig. 3a). Together, the results demonstrated a causal relationship between [Ca^2+^]_I_ and the strength of the Cx36–CaMKIIα interaction at GJPs.

[Ca^2+^]_I_ elevation is considered to be the critical step to initiate the binding of CaM to its CaMKIIα binding site, followed by binding of this complex to Cx36 (Fig. 3b). We used FRET to better understand how Ca^2+^ orchestrates short-range molecular changes of the Cx36–CaMKIIα complex at the GJP. Under normal cell growth conditions, Cx36–DsRed and CaMKIIα–ECFP co-expressed in Neuro2a cells showed significant FRET_eff_, which increased after treatment with Ca^2+^ ionophore (non-stimulated: 5.6 ± 0.5, *n* = 20; ionomycin: 9.9 ± 1.1, *n* = 19, *p* = 0.0059) (Fig. 3c). A low [Ca^2+^]_I_ condition was established with 24 µM BAPTA-AM, which abolished the interaction between Cx36 and CaMKIIα indicated by a FRET_eff_ of less than 1.7% (1.6 ± 0.3, *n* = 20, *p* = 6.0 × 10^−6^). Treatment with the solvent DMSO alone did not significantly alter Cx36–CaMKII FRET (DMSO: 6.6 ± 0.8, *n* = 23, *p* = 0.66) (Fig. 3c). The above treatments caused no significant differences in GJP size. When cells co-expressed Cx36 and CaMKIIα the GJP frequency was affected when [Ca^2+^]_I_ was chelated by BAPTA-AM (Supplementary Fig. S4a–c).

### NMDA receptor activation modulates Cx36 interaction with CaMKII

**Fig. 4:**
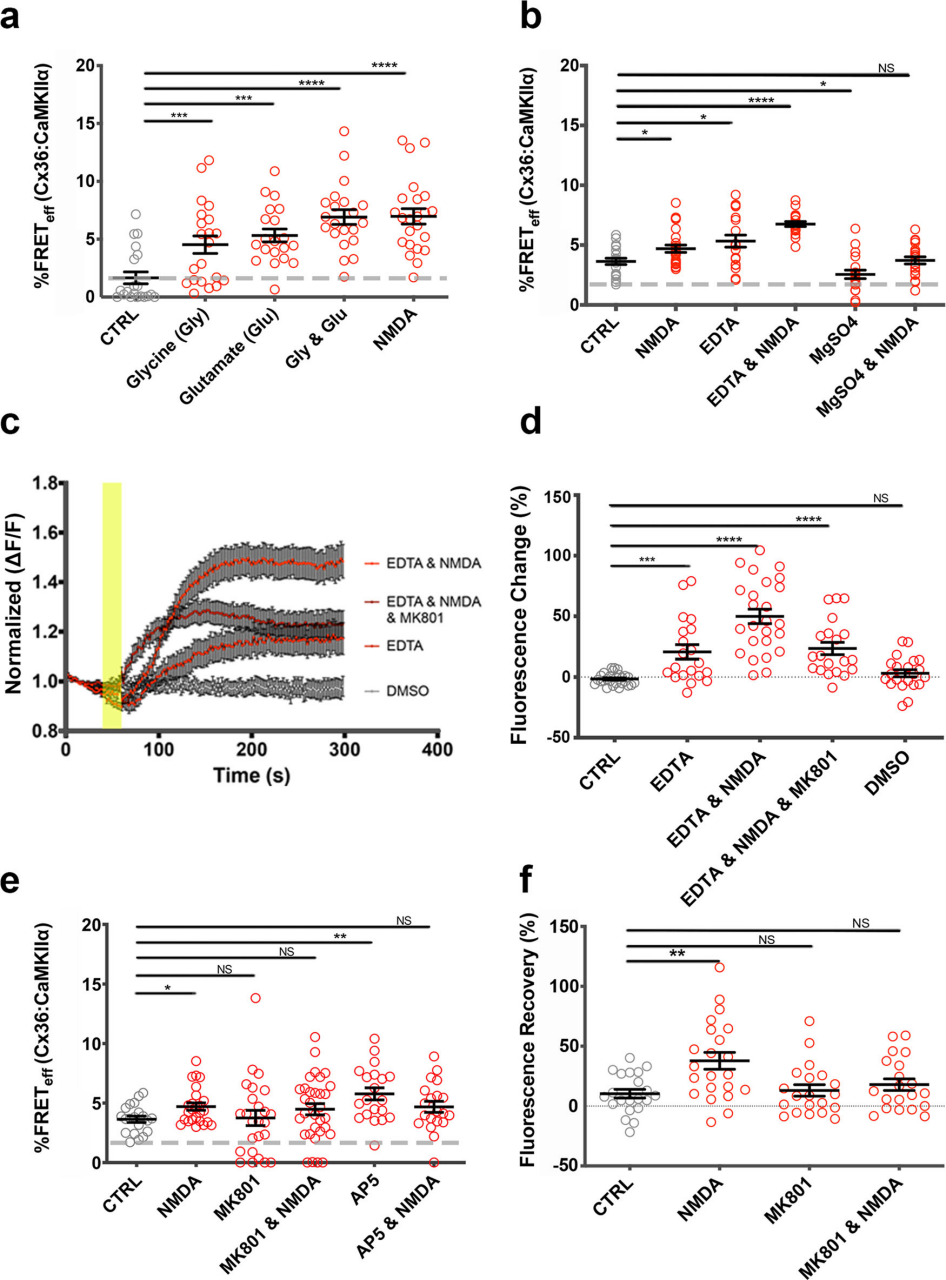
Impact of NMDA receptor activation on the modulation of the compactness of the Cx36–CaMKIIα interaction complex. In **a**, **b**, **e**, Neuro2a cells co-transfected with Cx36–DsRed and CaMKIIα–ECFP. The FRET efficiency (FRET_eff_) was determined at 48 h post-transfection. **a** FRET_eff_ after treatment with glycine (1 mM), glutamic acid (10 mM), and NMDA (50 µM). **b** FRET_eff_ after treatment with EDTA (3 mM), NMDA (50 µM), and MgSO_4_ (5 mM). **c**, **d** Neuro2a cells expressing Arclight S249 and Cx36–DsRed. **c** Arclight fluorescence change over 5 min with a yellow panel indicating the time window when compounds were added (*n* > 25, mean ± SEM). **d** Quantitative analysis of Arclight fluorescence changes in response to drug treatments. **e** FRET_eff_ following treatment with NMDA receptor antagonists MK801 and AP5. **f** Ethidium bromide uptake after photobleaching assay analysis of Cx36–EGFP expressing Neuro2a cells treated with NMDAR antagonists. Beeswarm graphs with mean ± SEM. Dashed line indicates the 1.7% limit of FRET; Mann–Whitney *U* significance (two-tailed), ****p < 0.0001, ***p < 0.001, **p < 0.01, *p < 0.05, NS not significant.

Neuro2a cells expressed low but detectable mRNA levels of multiple ionotropic NMDA receptor subunits and responded to NMDA stimulation (50 µM) with a rapid increase in [Ca^2+^]_I_ (Supplementary Fig. S5a, b; Supplementary Table S2). The response to NMDA was used to correlate receptor activation with structural changes of the Cx36–CaMKII interaction complex (Fig. 4). Stimulation of the NMDA receptors (NMDAR) with glycine (1 mM) and glutamate (10 mM) (Fig. 4a) caused a significant increase in FRET_eff_ of Cx36–DsRed and CaMKIIα–ECFP. Results imply that CaMKII is bound more tightly to Cx36, in a dose-dependent manner when compared to ionomycin treatment (Supplementary Fig. S5c) (non-stimulated: 1.7 ± 0.5, *n* = 20; glycine: 4.5 ± 0.8, *n* = 21, *p* = 6.3 × 10^−4^; glutamate: 5.3 ± 0.6, *n* = 20, *p* = 1.50 × 10^−4^; glycine and glutamate: 6.2 ± 0.5, *n* = 19, *p* = 6.79 × 10^−6^; NMDA: 7.0 ± 0.7, *n* = 23, *p* = 3.25 × 10^−6^). The agonist NMDA showed effects similar to the co-stimulation with glutamate and glycine.

Experimentally reduced extracellular magnesium (Mg^2+^) levels activate NMDARs allowing a surge of Ca^2+^ influx into the cell, whereas high Mg^2+^ levels limit the probability of NMDAR opening. Treatment with 10 µM EDTA to reduce Mg^2+^ levels led to an increase in FRET_eff_ that was similar to activation with NMDA (non-stimulated: 3.6 ± 0.3, *n* = 20; NMDA: 4.9 ± 0.4, *n* = 22, *p* = 0.02; EDTA: 5.3 ± 0.5, *n* = 19, *p* = 0.013). Removal of nonessential amino acids including glutamate and glycine from the cell growth medium abolished the increase in FRET_eff_ after EDTA treatment (Supplementary Fig. S6). Simultaneous addition of both EDTA and NMDA achieved the most substantial change in Cx36–CaMKIIα interaction (EDTA and NMDA: 6.8 ± 0.2, *n* = 19, (to CTRL) *p* = 2.0 × 10^−7^). Conversely, 5 mM MgSO_4_ treatment reduced FRET_eff_ when compared to control (MgSO_4_: 2.6 ± 0.4, *n* = 19, *p* = 0.02) and blocked potentiation upon NMDA stimulation (MgSO_4_ and NMDA: 3.7 ± 0.3, *n* = 20, *p* = 0.82) (Fig. 4b). The results indicate that extracellular Mg^2+^ and NMDA levels alter the compaction of Cx36–CaMKII, consistent with their activation of the NMDAR.

Membrane potential changes caused by activation of NMDA receptors were detected using the green fluorescent protein sensor Arclight S249 as a surrogate for electrophysiology. After chelating Mg^2+^ with EDTA alone, or in tandem with NMDA stimulation, membrane depolarization was detected by an increase in Arclight S249 fluorescence (Fig. 4c). The quantification of fluorescence changes is summarized in Fig. 4d (baseline (CTRL): −1.6 ± 0.9, *n* = 27; EDTA: 20.6 ± 5.9, *n* = 20; *p* = 2.15 × 10^−4^; EDTA and NMDA: 49.9 ± 6.0, *n* = 25, *p* = 2.0 × 10^−9^). The non-competitive NMDAR antagonist MK801 (dizocilpine) reduced the effects of both EDTA and NMDA significantly (EDTA and NMDA and MK801: 23.4 ± 5.1, *n* = 20, *p* = 2.0 × 10^−6^). The DMSO solvent control caused no fluorescence change (DMSO: 3.1 ± 2.9, *n* = 21; *p* = 0.1).

FRET measurements revealed the relationship between NMDAR activation and the binding of CaMKIIα to Cx36 after treatment with the NMDAR antagonists MK801 or AP5/APV (Fig. 4e, Supplementary Fig. S5d). MK801 targets the NMDA receptor pore formed by NR1 and NR2 subunits, and we found that MK801 treatment altered the proximity of the Cx36–DsRed and CaMKIIα–ECFP complex, but blocked the increased FRET due to NMDA stimulation (MK801: 3.3 ± 0.5, *n* = 24; *p* = 0.64; MK801 and NMDA: 4.5 ± 0.5, *n* = 32, *p* = 0.16). Targeting the NR2B binding site with the competitive antagonist D-AP5 (APV) had a similar effect, augmenting the basal FRET between Cx36–DsRed and CaMKIIα–ECFP but abolishing its response to NMDA activation (D-AP5: 5.8 ± 0.5, *n* = 20, *p* = 0.002; D-AP5 and NMDA-stimulated: 4.7 ± 0.5, *n* = 20, *p* = 0.06).

Next, an ethidium bromide uptake and recovery after photobleaching assay evaluated the impact of activation and inhibition of NMDA receptors on the gap junction function. Neuro2a cells expressing Cx36–EGFP and DsRed–CaMKII showed significant ethidium bromide redistribution after stimulation of NMDA receptors (non-stimulated: 10.3 ± 3.6, *n* = 21; NMDA: 37.8 ± 7.0, *n* = 22, *p* = 0.003). The pharmacological blockage of NMDARs with MK801 suppressed the ethidium bromide transfer through GJPs and inhibited the enhanced ethidium bromide transfer caused by NMDA (MK801: 13.1 ± 4.8, *n* = 20, *p* = 0.94; MK801 and NMDA: 17.9 ± 4.9, *n* = 20, *p* = 0.36 (Fig. 4f). The quantification of the GJP frequency and size showed a significant reduction in the number of gap junctions when NMDARs were blocked for 30 min with MK-801 or 10 min with AP5, suggesting that [Ca^2+^]_I_ changes can modulate gap junction communication rapidly (Supplementary Fig. S5e, f). The data establish NMDA receptors as a gateway for Ca^2+^ entry that enhances the binding of Cx36 to the CaM/CaMKII holoenzyme.

### Pannexin 1 modulates the interaction between Cx36 and CaMKII

**Fig. 5:**
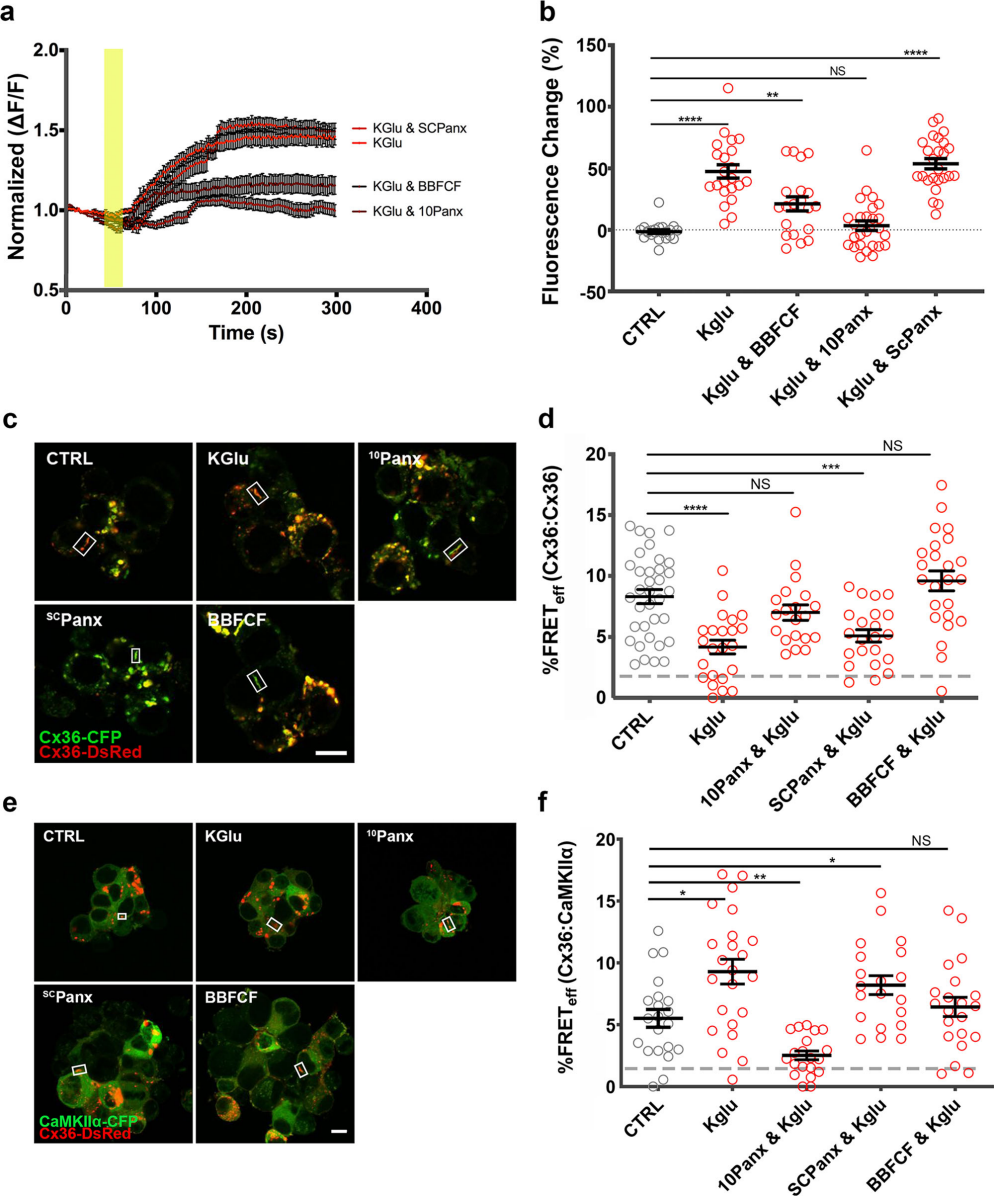
The activation of endogenous Panx1 modulates the interaction between Cx36 and CaMKII at the GJP. **a** Arclight fluorescence change over five minutes after treatment with KGlu, KGlu, and ^10^Panx, and KGlu and ^SC^Panx. The yellow panel indicates the time at which treatments were added (values: mean ± SEM). **b** Arclight fluorescence change quantification by comparing baseline (before one minute) to the final five minutes with different drug treatments. **c** Neuro2a cells double transfected with DsRed–Cx36 and ECFP–Cx36. GJPs are highlighted with rectangles. **d** FRET_eff_ in response to treatments at GJPs. **e** Neuro2a cells double transfected with DsRed–Cx36 and ECFP–CaMKIIα. GJPs are highlighted with rectangles. **f** Treatments altered FRET_eff_ of Cx36–DsRed and Cx36–ECFP WT and various blockers with and without 40 μM potassium gluconate (KGlu). Beeswarm graphs in **d**, **f** with mean ± SEM; dashed line indicates the 1.7% or 10 nm limit of FRET; Mann–Whitney *U* significance (two-tailed), ****p < 0.0001, ***p < 0.001, **p < 0.01, *p < 0.05, NS not significant; scale bars in **c**, **e** = 10 µm).

Neuro2a cells express low levels of Panx1 and respond to potassium gluconate stimulation (40 mM KGlu)^25^, as shown here using the genetically encoded membrane potential probe Arclight S249 (Fig. 5a, b) (baseline (CTRL): −1.5 ± 1.5, *n* = 22; KGlu: 47.4 ± 5.4, *n* = 22; *p* = 3.0 × 10^−8^). The fluorescence of Arclight S249 in the presence of KGlu was reduced when Panx1 channels were blocked with the blue food dye (BB-FCF), a selective inhibitor of Panx1 when used in Neuro2a cells^26^, or the ^10^Panx peptide^27^ (compared to CTRL; BB-FCF: 21.1 ± 5.7, *n* = 20; *p* = 0.0038; ^10^Panx: 3.4 ± 3.9, *n* = 26; *p* = 0.66). The scrambled control peptide ^SC^Panx had no effect (^SC^Panx: 53.7 ± 4.2, *n* = 25; *p* = 1.13 × 10^−8^).

Treatment of Neuro2a cells co-transfected with Cx36–ECFP and Cx36–DsRed with 2 mM [Ca^2+^]_E_ and 40 mM KGlu decreased FRET_eff_ at GJPs, indicating Cx36 channel opening (non-stimulated: 8.5 ± 0.7, *n* = 28; KGlu: 4.2 ± 0.6, *n* = 23, *p* = 4.9 × 10^−5^) (Fig. 5c, d). The Panx1 channel blockers, ^10^Panx peptide or BB-FCF, significantly reduced the effects of KGlu stimulation, while the scrambled version of ^10^Panx (^SC^Panx) had no noticeable effect (^10^Panx and KGlu: 7.0 ± 0.6, *n* = 20, *p* = 0.14; ^SC^Panx and KGlu: 5.1 ± 0.5, *n* = 22, *p* = 9.3 × 10^−4^; BB-FCF and KGlu: 9.6 ± 0.8, *n* = 24, *p* = 0.16).

KGlu stimulation increased FRET_eff_ at GJPs significantly when Neuro2a cells co-expressed CaMKIIα–ECFP and Cx36–DsRed (non-stimulated: 5.1 ± 0.4, *n* = 21; K-Glu: 9.3 ± 1.0, *n* = 23, *p* = 0.014) (Fig. 5e, f). The effect of KGlu was suppressed with ^10^Panx and BB-FCF (^10^Panx and K-Glu: 2.5 ± 0.4, *n* = 20, *p* = 0.001; BB FCF and KGlu: 6.4 ± 0.8, *n* = 21, *p* = 0.38), but was unchanged by ^SC^Panx treatment (^SC^Panx and K-Glu: 8.2 ± 0.8, *n* = 20, *p* = 0.02). This result suggested that Panx1 channels modulate the binding of Cx36 to CaMKII.

### Overexpression of Panx1 rescued the Cx36–CaMKII interaction in Panx1 knockdown cells

**Fig. 6:**
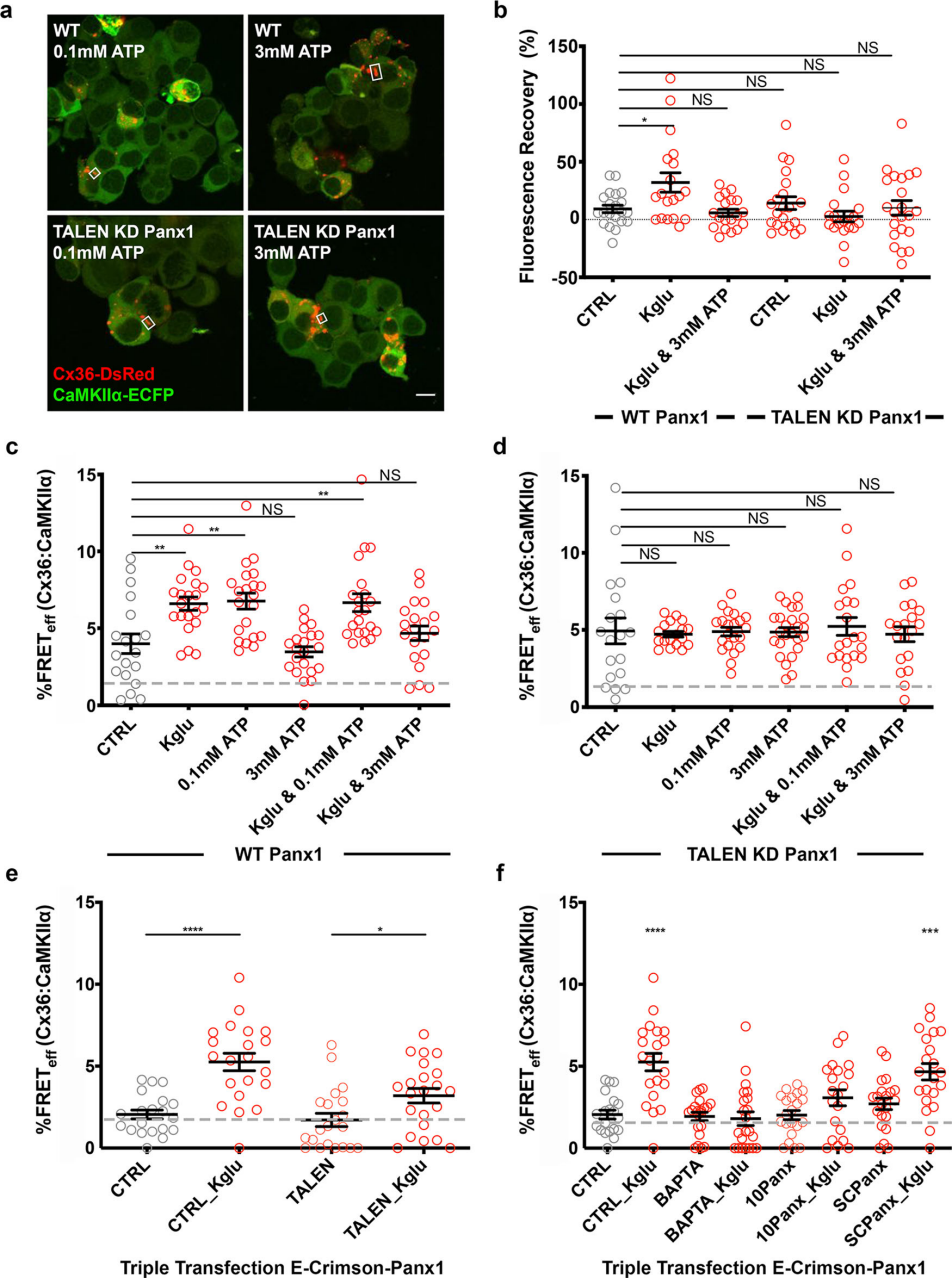
Knocking down Panx1 channel expression in Neuro2a cells or blocking its function reduced the compaction of Cx36–CaMKII in response to KGlu and low ATP concentration. Neuro2a cells (WT and TALEN KD Panx1) were either transfected doubly with Cx36–DsRed and CaMKIIα–ECFP or singly transfected with Cx36–EGFP for the ethidium bromide recovery after photobleaching assay. **a** Normal GJP formation in both WT and KD Panx1 cells (GJPs indicated by white boxes; scale bar = 10 µm). **b** Ethidium bromide recovery after photobleaching assay of WT Neuro2a cells or KD Panx1 cells treated with potassium gluconate (KGlu) or 3 mM ATP. **c**, **d** FRET analysis of double transfected WT and KD Panx1 cells treated with KGlu, or 0.1 or 3 mM ATP. **e** Triple transfected CTRL or KD Panx1 cells with Panx1- E-Crimson were analyzed with and without KGlu. **f** Triple transfected cells were analyzed after being treated with BAPTA, ^10^Panx, or ^SC^Panx with or without KGlu. Beeswarm graphs with mean ± SEM; dashed line indicates the 1.7% limit of FRET; Mann–Whitney *U* significance (two-tailed), ****p < 0.0001, ***p < 0.001, **p < 0.01, *p < 0.05, NS not significant.

A Neuro2a cell line with a targeted knockdown of Panx1 (KD-Panx1) was generated using TALEN technology. KD-Panx1 cells have a 17-base pair (bp) deletion eliminating the start codon of the Panx1 protein. The cells did not show ethidium bromide dye-uptake after KGlu stimulation (Supplementary Fig. S7a–c; Supplementary Methods). Neuro2a cells expressing Cx36 and endogenous Panx1 showed enhanced ethidium bromide uptake and recovery after photobleaching and KGlu stimulation that was blocked by ATP (non-stimulated wild-type CTRL: 9.2 ± 3.2, *n* = 21; KGlu: 32.1 ± 8.4, *n* = 19, *p* = 0.04; KGlu and 3 mM ATP: 5.8 ± 3.1, *n* = 19, *p* = 0.44). Neither the increased coupling or the block by ATP were observed in TALEN KD-Panx1 cells when compared to wild-type CTRL (TALEN KD-Panx1 non-stimulated: 14.2 ± 5.5, *n* = 21, *p* = 0.95; KGlu: 2.63 ± 4.6, *n* = 19, *p* = 0.09; KGlu and 3 mM ATP: 10.1 ± 6.3, *n* = 22, *p* = 0.92) (Fig. 6b).

The high/low ATP paradigm for closing/opening Panx1 channels^28,29^ was used to demonstrate the impact of Panx1 on the CaMKIIα–Cx36 interaction. In Neuro2a cells, FRET_eff_ of Cx36–CaMKIIα increased with KGlu treatment (wild-type CTRL non-stimulated: 4.0 ± 0.6, *n* = 19; KGlu: 6.6 ± 0.4, *n* = 21, *p* = 0.0028), 0.1 mM ATP (6.8 ± 0.5, *n* = 21, *p* = 0.0033) or both (0.1 mM ATP and KGlu: 6.5 ± 0.6, *n* = 22, *p* = 0.0021), while 3 mM ATP showed a reduction in FRET_eff_, both without (3.5 ± 0.3, *n* = 21, *p* = 0.80) and with KGlu (4.7 ± 0.5, *n* = 20, *p* = 0.26) (Fig. 6c). KD-Panx1 cells showed no response of the Cx36–CaMKIIα complex to any of these treatments compared to wild-type CTRL (KD-Panx1 CTRL non-stimulated: 4.9 ± 0.8, *n* = 19; KGlu: 4.7 ± 0.2, *n* = 18, *p* = 0.54; 0.1 mM ATP (4.9 ± 0.3, *n* = 21, *p* = 0.43); 3 mM ATP (4.9 ± 0.3, *n* = 25, *p* = 0.44); 0.1 mM ATP and KGlu (5.2 ± 0.6, *n* = 20, *p* = 0.40), or 3 mM and KGlu (4.7 ± 0.5, *n* = 19, *p* = 0.56) (Fig. 6d). The responsiveness to KGlu was at least in part rescued by transfecting Panx1–Crimson in both WT (CTRL with Panx1–Crimson non-stimulated: 2.1 ± 0.3, *n* = 20; CTRL with KGlu: 5.3 ± 0.5, *n* = 21, *p* = 4.0 × 10^−5^) and KD-Panx1 cells (TALEN with Panx1–Crimson non-stimulated: 1.72 ± 0.4, *n* = 21; TALEN with KGlu: 3.2 ± 0.4, *n* = 22, *p* = 0.02) (Fig. 6e; Supplementary Fig. S8).

We speculated that overexpression of Panx1 channels provided a condition of Ca^2+^ entry and that blocking Panx1 with ^10^Panx or chelating Ca^2+^ with BAPTA-AM should attenuate the effects. Indeed, baseline treatment with ^10^Panx or BAPTA-AM abolished any significant FRET_eff_, indicating interaction between CaMKIIα and Cx36, whereas the scrambled control Panx1 peptide had no effect (wild-type CTRL: 2.1 ± 0.3, *n* = 20; ^10^Panx: 2.0 ± 0.3, *n* = 21, *p* = 0.9; ^SC^Panx: 2.7 ± 0.4, *n* = 21, *p* = 0.14; BAPTA-AM: 1.9 ± 0.3, *n* = 21, *p* = 0.94). The scrambled control Panx1 peptide did not block KGlu induced interaction between CaMKIIα and Cx36 (^SC^Panx and KGlu: 4.7 ± 0.5, *n* = 21, *p* = 1.8 × 10^−4^), and FRET_eff_ was not changed when KGlu was added in the presence of ^10^Panx or BAPTA-AM (^10^Panx and KGlu: 3.1 ± 0.5, *n* = 20, *p* = 0.12; BAPTA-AM and KGlu: 1.8 ± 0.4, *n* = 22, *p* = 0.25) (Fig. 6f).

### The adoption of a compact structure of the Cx36–CaMKII interaction complex is modulated by an NMDA receptor—Src family kinase—Panx1 signaling pathway

**Fig. 7:**
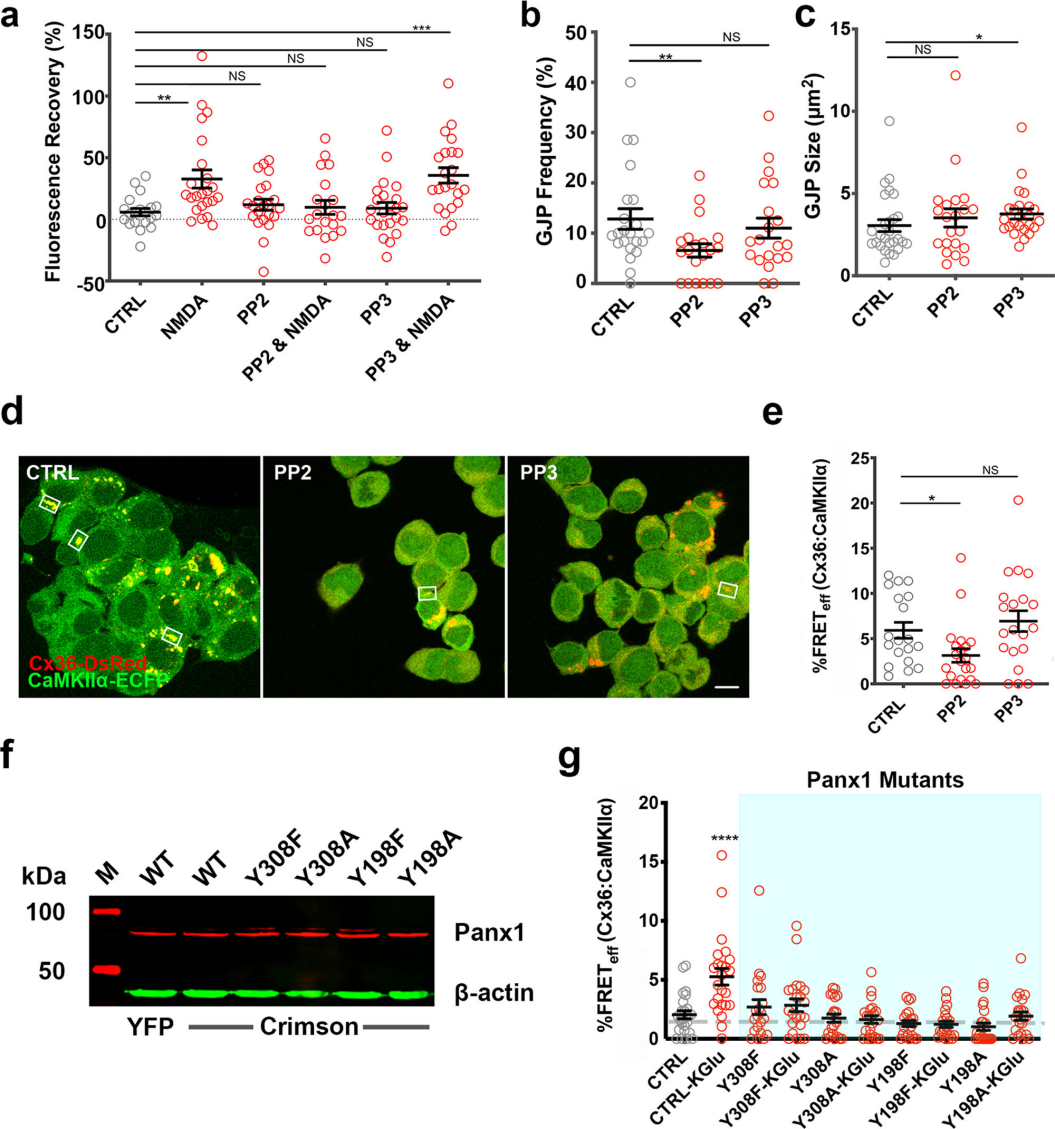
A Src-family kinase is involved in modulating the Cx36–CaMKII interaction. **a** Ethidium bromide recovery after photobleaching assay showed fluorescence recovery of ethidium bromide through Cx36–EGFP gap junctions under the effects of PP2 or PP3 with or without NMDA stimulation. **b**, **c** Quantification of gap junction frequency and size with the addition of PP2 or PP3. **d** Neuro2a cells expressing Cx36–DsRed and CaMKIIα–ECFP showing normal gap junction expression with PP2 or PP3. **e** FRET_eff_ of Cx36–DsRed and CaMKIIa–ECFP under the effects of PP2 or PP3. **f** Western blot confirming expression of Panx1 WT and mutants (Y308F, Y308A, Y198F, and Y198A) tagged with EYFP or E-Crimson in 48 h post-transfected Neuro2a cells (Panx1 = red; β-actin = green). **g** FRET_eff_ of Cx36 with CaMKII interaction in triple transfected (Cx36–DsRed, CFP–CaMKII, and Panx1–E-Crimson (WT, Y308F, Y308A, Y198F, and Y198A) Neuro2a cells upon KGlu stimulation. Beeswarm graphs with mean ± SEM; dashed line indicates the 1.7% limit of FRET; Mann–Whitney *U* significance (two-tailed), ****p < 0.0001, ***p < 0.001, **p < 0.01, *p < 0.05, NS not significant.

Initial experiments were directed towards Ca^2+^ entry through ionotropic NMDA receptors and how this affects CaMKII–Cx36 interaction. In a separate investigation line, we turned to whether post-translational modification of Panx1 by SFKs affects CaMKII–Cx36 interaction and increases coupling after stimulation of ionotropic NMDA receptors. This change in focus was motivated by research demonstrating a signaling pathway between metabotropic NMDA receptors and Panx1, and the regulation of Panx1 channel function by SFK-mediated phosphorylation at the two tyrosine residues Y308 and Y198^11^. In ethidium bromide uptake and recovery after photobleaching experiments, activation of Cx36 GJC by stimulation of the NMDA receptor increased the redistribution of ethidium bromide between Neuro2a cell pairs after photobleaching (non-stimulated: 5.8 ± 3.0, *n* = 20; NMDA: 32.7 ± 7.4, *n* = 23, *p* = 0.0014) (Fig. 7a). The increase in dye in the photobleached regions of interest below GJPs was suppressed by co-application of the Src kinase inhibitor 4-amino-5-(4-chlorophenyl)-7-(t-butyl)pyrazolo[3,4-d]pyrimidine (PP2) (PP2: 11.8 ± 4.5, *n* = 22, *p* = 0.15; PP2 and NMDA: 9.7 ± 5.7, *n* = 20, *p* = 0.89). The negative control 4-amino-1-phenyl-1H-pyrazolo[3,4-d] pyrimidine (PP3) had no effect on ethidium bromide redistribution (PP3: 9.0 ± 4.7, *n* = 23, *p* = 0.76; PP3 and NMDA: 35.1 ± 6.2, *n* = 22, *p* = 2.6 × 10^−4^). Gap junction frequency (CTRL: 12.8 ± 2.0, *n* = 22; PP2: 6.6 ± 1.3, *n* = 20, *p* = 0.006; PP3: 11.0 ± 2.0, *n* = 20, *p* = 0.34), but not the size of GJPs was affected by PP2 treatment (CTRL: 3.1 ± 0.4, *n* = 22; PP2: 3.5 ± 0.6, *n* = 21, *p* = 0.66; PP3: 3.8 ± 0.3, *n* = 24, *p* = 0.02) (Fig. 7b, c). Double transfected Neuro2a cells treated with PP2 and PP3 showed regular cell morphology (Fig. 7d).

SFKs also modulated the compactness of the Cx36–DsRed and CaMKIIα–ECFP complex. Treatment with PP2 reduced FRET_eff_ to control levels, while PP3 was not effective (CTRL: 5.9 ± 0.9, *n* = 19; PP2: 3.1 ± 0.8, *n* = 21; *p* = 0.013; PP3: 6.9 ± 1.1, *n* = 20, *p* = 0.55) (Fig. 7e).

FRET_eff_ of the Cx36–CaMKII interaction complex in KD-Panx1 cells was significantly increased after KGlu stimulation only when Panx1–E-Crimson was overexpressed (non-stimulated: 2.0 ± 0.3, *n* = 25; KGlu: 5.3 ± 0.7, *n* = 24, *p* = 7.5 × 10^−5^). Expression of mutants, Panx1–Y308F and Panx1–Y198A, showed no rescue of the phenotype (Panx1–Y308F: 2.6 ± 0.6, *n* = 22, *p* = 0.65; Panx1–Y308F and KGlu: 2.85 ± 0.54, *n* = 22, *p* = 0.46; Panx1–Y308A: 1.75 ± 0.35, *n* = 21, *p* = 0.53; Panx1–Y308A and KGlu: 1.63 ± 0.32, *n* = 22, *p* = 0.46). The Panx1–Y198F and –Y198A mutants reduced Cx36–CaMKII interaction (Panx1–Y198F: 1.29 ± 0.25, *n* = 21, *p* = 0.11; Panx1–Y198F and KGlu: 1.22 ± 0.26, *n* = 21, *p* = 0.08; Panx1–Y198A: 1.03 ± 0.31, *n* = 23, *p* = 0.01; Panx1–Y198A and KGlu: 1.92 ± 0.35, *n* = 22, *p* = 0.78) (Fig. 7f, g). The results demonstrated the role of SFK phosphorylation of Panx1 in modulating the interacting complex of Cx36 and CaMKII.

## Discussion

The activity-dependent strengthening or weakening of chemical synaptic transmission is regarded as a fundamental mechanism by which neural circuits are formed or broken. A principal molecular mechanism by which excitatory synapses are strengthened is through the interaction of CaMKII and NMDA receptor subunits. Evidence for similar use-dependent facilitation of electrical synaptic strength was first revealed at auditory mixed synapses on the goldfish Mauthner cells^30,31^. Like chemical synaptic potentiation, this facilitation was shown to depend on CaMKII binding to the channel protein, in this case, one or more teleost homologs of Cx36^32^. We previously demonstrated that the mammalian neuronal gap junction protein Cx36 also exhibits use-dependent facilitation, the so-called “run-up”^7,33^. The Cx36 protein interacts with and is phosphorylated by CaMKII in vitro, similar to the CaMKII interaction with the NR2B subunit of ionotropic NMDA receptors^6^. We further showed that phosphorylation by CaMKII strengthens junctional currents of Cx36 channels, thereby conferring functional plasticity on electrical synapses formed by this protein^7^.

Here, using neuroblastoma cells as an efficiently manipulatable neural cell line, we identify components of a signaling pathway that modifies the CaMKII interaction with Cx36 at the GJP. The signaling pathway consists of ionotropic NMDA receptors, SFK, and Panx1 and is distinct from protein interactions that we and others have previously reported involving Cx36. These include binding to Ca^2+^ loaded CaM^34^, which mainly occurs at the ER/Golgi complex^18^, the interaction with tubulin facilitating microtubule-dependent transport to the GJP^24^, or the removal of Cx36 from the GJP by binding to caveolin-1^35^. Our present interpretation is that these known interactions primarily contribute to on-demand transport to the GJP and endocytosis of Cx36.

The findings reported here were accomplished with FRET measurements of fluorescent protein-tagged construct pairs as a sensitive method to detect local pathways of protein interactions at the cell membrane. This method revealed increased compact conformation of protein–protein complexes, interpreted as tighter interactions between the pairs, when [Ca^2+^]_I_ was elevated either with Ca^2+^ ionophore or by activation of ionotropic NMDA receptors. Interestingly, both GJP size and frequency have been reduced after the loss of CaM/CaMKII binding or when NMDARs were blocked. These observations suggest multi-faceted roles of CaMKII including the transport/insertion/removal of Cx36 at GJPs, resembling a potential similarity to feedback mechanisms found at NMDARs. CaMKII phosphorylation of NMDARs plays a central role in controlling the number and activity of the receptors and determining the strength of excitatory synaptic transmission^36^. The results made in our minimalistic neuroblastoma cell model provide mechanistic insight into how a critical function of electrical synapses is modulated by crosstalk with molecular components that are generally hallmarks of chemical synapses. Neuronal gap junctions are often found in close proximity to glutamatergic synapses, where they can be considered as functional mixed synapses^2^. Using established methods of manipulating NR1/NR2B subunit activation (by relieving the magnesium block^37,38^, inhibition or activation of extracellular receptor sites, or pharmacological modulation^39,40^), we found that ionotropic NMDA receptors constitute an important entry point for [Ca^2+^]_E_. The increase in [Ca^2+^]_I_ was identified as an essential driver of the transition of the Cx36–CaMKII complex to a more compact state.

Since the 90s, electrophysiological studies in lower vertebrates demonstrated NMDA receptor-mediated strengthening of electrical coupling between neurons in mixed synapses^31,41^ and implicated CaMKII^42^. Later, regulation and plasticity of electrical transmission by NMDA receptor-initiated sequence of events were found in the mammalian brain in synaptic loci and at extra-synaptic loci proximal to gap junctions^43^. Our previous report of “run-up plasticity” has demonstrated that Cx36 binds Ca^2+^-activated CaMKII in vitro, using binding domains with similarities to the binding sites found in the NR2B subunit of the NMDA receptor. We concluded that this was evidence for a shared molecular basis of use-dependent plasticity^6,7^. The results identify short-range structural rearrangements which increase the adoption of a compact state of the Cx36–CaMKII holoenzyme complex as a fundamental requirement to open Cx36 channels. We predict that recent advances in cryoEM technologies provide an opportunity to solve the differences of compact and open states of the Cx36–CaMKII complex at an atomic resolution.

An additional way in which activation of the signaling complex may be modulated is through intracellular Mg^2+^, which was shown by Palacios-Prado et al.^44^ to inhibit Cx36-dependent gap junctional intercellular communication in cell lines exogenously expressing Cx36, and in brain slice recordings from neurons in the mesencephalic nucleus known to express endogenous Cx36. The authors argued that Mg^2+^ concentration changes sufficient to alter coupling might occur physiologically by ATP fluctuations, which reduces Mg^2+^ and thus increase coupling. It was suggested that the effect of Mg^2+^ on coupling strength was due to direct interaction with the channel. However, Mg^2+^ also competes with Ca^2+^ for binding to calmodulin^45^, and the effects reported in that study are consistent with an interference with the step-wise assembly of the Cx36–CaMKII signaling complex.

The involvement of Panx1 in regulating Cx36 was unexpected. To our knowledge, this is the first observation of functional interaction between members of the connexin and pannexin gene families. Previously, several stimuli have been reported to activate Panx1 channels^46–48^, including ionotropic NMDARs activated during anoxia or following energy deprivation^49,50^. The Thompson group also reported that Panx1 activation required SFKs, which corroborated that the link between NMDARs and Panx1 were SFKs^12^. The discovery that the activation of ionotropic NMDA receptors, Panx1, and SFKs increase intracellular calcium levels, which drive Cx36 bound to CaMKII to an open state, demonstrates molecular and perhaps mechanistic interaction between chemical and electrical communication in nerve cells. They also suggest a possible involvement of Cx36 in the postsynaptic metabotropic NMDARs, Panx1, and SFKs signaling complex reported by the Thompson group.

In primary neurons, a signaling pathway involving ionotropic NMDA receptors and Panx1 is expected to be restricted to the postsynaptic cell membrane based on known protein localization data. Ionotropic NMDA receptors are located close to Cx36 at mixed synapses in excitatory neurons^1,2^. Panx1 proteins were found in rodent hippocampal and cortical principal neurons accumulating at high concentration in proximity to postsynaptic densities^9^. In contrast, CaMKII and SFKs can be found in both pre- and postsynaptic compartments. The restricted localization of both NMDA receptors and Panx1 in postsynaptic compartments suggests that the signaling complex in primary central nervous system neurons is not a simple mirror image across the GJP. This finding adds to growing evidence for functional asymmetry caused by differences in post-translational modifications of individual Cx36 proteins^51^, or in the complement of Cx36-associated proteins found at the electrical synapse density^36,52,53^. Taken together, the outcomes of this study add to the best of our knowledge a new twist to how electrical and chemical synapses communicate. They suggest that interactions between the two modes of neuronal communication exist, are direct, interdependent, and might facilitate synchrony and adaptive plasticity at mixed synapses.

## Methods

### Cell culture, transient transfection, and western blot

Neuroblastoma 2a (Neuro2a) cells were cultivated in DMEM with 2 mM glutamine, 1% nonessential amino acids (NEAA), 1% penicillin, and streptomycin (PS), and 10% fetal bovine serum at 37 °C and 5% CO_2_ in a humid-controlled incubator. Cells were grown in plastic 24-well plates or glass-bottom dishes (MatTek Corporation, Ashland, MA, USA) and transfected with 200 ng (single transfection), 400 ng (double transfection), or 600 ng (triple transfection) total endotoxin-free plasmid DNA, with the Effectene transfection protocol (Qiagen Inc., Valencia, CA, USA). All plasmids used in this study were generated by site-directed mutagenesis (Supplementary Methods; Supplementary Table S2). For western blot analysis, protein lysates were prepared 48 h post-transfection. Approximately 20 µg of protein was separated by 10% sodium dodecyl sulphate-polyacrylamide gel electrophoresis, transferred to 0.2 µm Midi format nitrocellulose membrane, and processed using the iBind^TM^ Western System (Bio-Rad Inc., Mississauga, ON, Canada). Primary antibodies were diluted 1:500 (mouse anti-GFP, Roche; rabbit anti-GFP (FL), Santa Cruz Biotechnologies, TX, USA; rabbit anti-Panx1, Invitrogen, 488100) and 1:15,000 (mouse anti-β-actin; Sigma-Aldrich Chemie GmbH, Munich, Germany). The secondary antibodies (LI-COR Biosciences, St. Lincoln, NE, USA) were diluted 1:15,000 (donkey anti-rabbit IRDye680LT) or 1:20,000 (goat anti-mouse IRDye800CW). Fluorescent signals were detected with the Odyssey^®^ CLx Infrared Imaging System using default settings (LI-COR Biosciences).

### TALEN Panx1 KD cells

Transcription Activator-Like Effector Technology (TALEN) cloning protocols were carried out described by Reyon and colleagues^54^. The ZiFit Targeter software (http://zifit.partner.org/ZiFiT/) was used to design constructs targeting a single NcoI restriction site located close to the start of the coding region in exon 1 of the mouse Panx1 gene (Supplementary Fig. S7a) (Ensembl: ENSMUSG00000031934). Constructs were assembled in the expression vectors JDS74 and JDS78 using the protocol introduced by Reyon and colleagues^54^. Neuro2a cells were transfected with both TALEN constructs at an equimolar ratio, followed by treatment with the nucleoside antibiotic blasticidin (10 μg/ml) for 2 weeks. Stable cell lines were established by using a limited dilution protocol in which single-cell suspensions of blasticidin-resistant cells were repeatedly (3×) diluted and expanded from individual cell colonies. Upon reaching 80–100% confluency, genomic DNA was extracted using the GeneJET Genomic DNA purification kit (Thermo Fisher Inc., Mississauga, ON, Canada). Polymerase chain reaction (PCR) amplification of the genomic DNA followed by restriction digest of a 226 bp amplicon with NcoI to confirm the loss of the NcoI site at the start of the Panx1 open reading frame. PCR products were cloned into pJET1.2 plasmid (Thermo Fisher Inc., Mississauga, ON, Canada) and sequenced for confirmation of indel mutations. A cell line with a 17 bp deletion at the start codon of the Panx1 protein was selected for further analysis (Supplementary Fig. S7).

### FRET analysis

Protein interactions at GJPs were investigated using the acceptor bleach protocol^18,21^. Neuro2a cells were transfected with DsRed-monomer and EGFP/ECFP-tagged expression vectors. Baseline readings were established before initiating the acceptor bleach protocol (five initial readings). In this protocol, DsRed-tagged proteins were photobleached using the 555 nm laser line at 100% intensity, while the intensity change of CFP or GFP tagged proteins was recorded using the 405 or 455 nm laser lines respectively. The experimental protocol was terminated upon reaching 10% of the acceptor channel baseline intensity. FRET efficiency was then calculated by using the FRET efficiency equation 1

$${\rm{FRET}}\,{\rm{efficiency}}\left(E \% \right)=\frac{({D}_{\rm{Post}}-{D}_{\rm{Pre}})}{{D}_{\rm{Post}}}$$

where *D*_post_ is the average intensity after bleaching, and *D*_pre_ is the average intensity before the bleach after subtracting the values of the background (noise).

### Ethidium bromide recovery after photobleaching assay

The assay has been reported previously^18,25^. Here, this assay was used to correlate functional changes with structural rearrangements of the GJP in the presence of stimulants or blockers. Transfected Neuro2a cells cultured for 48 h in 3.5 cm MatTek cell culture dishes were incubated with 10 μM of ethidium bromide in supplemented growth medium for 10 min at 37 °C and 5% CO_2_ prior to imaging_._ Transfected cells in MatTek dishes were placed in a live-cell imaging chamber (regulated at 37 °C and 5% CO_2_) and imaged with Zeiss 700 confocal microscope. Cell pairs expressing Cx36–EGFP were selected, and a time-lapse baseline image was recorded (defined as 100%). One cell of each cell pair was bleached using the 555 nm laser line with 100% laser power intensity (Supplementary Fig. S2a). The number of iterations (~40 iterations at 100% intensity) in the bleaching protocol was terminated upon achieving a 40–50% reduction of baseline fluorescence. A voxel of 36.2 μm × 36.2 μm × 1.6 μm was bleached using a non-confocal imaging mode. The recovery of ethidium bromide fluorescence in the center plane of this voxel after bleaching was measured over time (180 s) in confocal imaging mode. In all experiments, three regions of interest (ROI: C1, C2, R) were selected for analysis. C1 was placed outside the cell pair to determine the background fluorescence. ROIs (R) were placed inside the bleached cell close (0.5–1 μm distance to the GJP; Supplementary Fig. S2b), and distant (C2; by the cell membrane) to the GJP. The fluorescence recovery in percent was calculated using Eq. 2

$${\rm{Fluorescence}}\ {\rm{recovery}}\ (\%)=\frac{({{F}}_{{\rm{average}}(3\,{{\min }}\ {\rm{post}}\ {\rm{bleach}})}-{{F}}_{\rm{bleach}})}{({{F}}_{{\rm{average}}\ ({\rm{pre}}\ {\rm{bleach}})}-{{F}}_{\rm{bleach}})}$$

### Ca^2+^ imaging

Intracellular Ca^2+^ was imaged in transfected Neuro2a cells that were incubated in DMEM^(−)phenol red^ with Ca^2+^ indicator Oregon Green 488 BAPTA-AM Cell Permeant (OGB) (Thermo Fisher Inc., Mississauga, ON, Canada) for 30 min on MatTek plates. Cells were washed with PBS^+/+^ before incubating for another 30 min in DMEM^(−)phenol red^. Cell pairs were imaged using a Zeiss Observer Z1 Spinning Disk Confocal Microscope equipped with a live-cell imaging chamber set to 37 °C and 5% CO_2_. Evolve TIRF camera with a Plan-Apochromat 63×/1.4 Oil DIC M27 objective was used to measure the Ca^2+^ signal changes over 300 cycles at 1-s intervals. The 488 nm laser line at 20% laser intensity was used to measure the cells. Experimental drug treatments were delivered after the first 10 cycles. Images were processed and analyzed in ImageJ with the Heatmap Histogram and Time Series Analyzer V3 plugin after background subtraction. Images were pseudo-colored using ImageJ’s Thermal LUT.

### Arclight S245 fluorescent voltage probe imaging

Membrane potential changes in double transfected Neuro2a cells expressing Cx36–DsRed and Arclight S245^55–57^ were imaged 48 h post-transfection. Cells were imaged using a Zeiss Observer Z1 Spinning Disk Confocal Microscope equipped with a live-cell imaging chamber set to 37 °C and 5% CO_2_. Evolve TIRF camera with a Plan-Apochromat 63×/1.4 Oil DIC M27 objective was used to measure the fluorescence change of Arclight S245 over 300 cycles at 2-s intervals. The 488 and 561 nm laser lines were at 15% and 10% laser intensity during the experiments. The first 30 cycles were used to establish the baseline for each experimental run, while drug treatments were delivered through tubing with a syringe during the 31st–40th cycle. The Definite Focus module of the microscope was used to maintain the plane of view throughout the experiment. Images were processed and analyzed in ImageJ with the Time Series Analyzer V3 plugin after background subtraction.

The fluorescence scaled to baseline was calculated using the Eq. 3

$${\rm{Fluorescence}}\ ({\rm{scaled}})=\frac{{F_{{\rm{time}}}}}{{F_{{{\rm{average}}\ ({\rm{baseline}})}}}}$$

and the fluorescence change (Δ*F*/*F*) was calculated using Eq. 4

$${\rm{Fluorescence}}\ {\rm{change}}\ (\Delta {F}/{F})=\frac{({{F}}_{{\rm{average}}\ (300)}-{{F}}_{{\rm{average}}\ ({\rm{baseline}})})}{({{F}}_{{\rm{average}}\ ({\rm{baseline}})})}$$

where *F*_time_ is the fluorescence at the given time, *F*_average (baseline)_ is the average of fluorescence in the first 30 cycles and *F*_average (300)_ is the average of fluorescence in the last 30 cycles to 5 min.

### Pharmacology

Before imaging, transfected cells were treated with different pharmacological agents to a final concentration in complete growth medium: BAPTA-AM (24 µM; 10 min; Thermo Fisher), ionomycin (2 µM; 5 min), NMDA (50 µM; 5 min), glutamate and glycine (10 and 2 mM; 1 min), EDTA (10 µM; 5 min), MgSO_4_ (5 mM; 10 min), MK801 (20 µM; 30 min), AP5 (100 µM; 10 min), PP2 (10 µM; 30 min), PP3 (10 µM; 30 min), ^10^Panx, scrambled control ^SC^Panx1 (100 µM, 10 min), all at 37 °C and 5% CO_2_. The final solvent concentration of DMSO was 0.002–0.02%. All reagents were obtained from Sigma-Aldrich.

### Statistics and reproducibility

Statistical analysis and data presentation were performed using the IBM-SPSS and R-software packages. Data were presented as (mean ± SEM). All data were analyzed with the Shapiro–Wilk normality test and Levene’s homogeneity of variance test. Data with normal and equal invariance were subjected to independent *t*-tests, and non-normal distributed data were subjected to Mann–Whitney *U* (2-tailed) tests. Outliers were determined and shown on figures but have been excluded from statistical analyses. The sample sizes (*n*) reported corresponding to cells or cell pairs as indicated. The minimum number of independent experimental replicates was *n* ≥ 3.

### Reporting summary

Further information on research design is available in the Nature Research Reporting Summary linked to this article.

## Supplementary information

Supplementary Information

Description of Supplementary Files

Supplementary Data 1

Reporting Summary

## Data Availability

All data generated or analyzed during this study are included in this published article (and its Supplementary Information file), Source data for Figs. 1–7, and Supplementary Figs. S1–S73 can be found in Supplementary Data 1. Raw image files will be made available by the corresponding authors upon reasonable request.
