# Supplementary material for: Convergent NMDA receptor—Pannexin1 signaling pathways regulate the interaction of CaMKII with Connexin-36
Source: Commun Biol. 2021 Jun 8;4:702. doi: 10.1038/s42003-021-02230-x (PMC8187354; doi:10.1038/s42003-021-02230-x)
Supplement: Supplementary file 1 — Supplementary Information [file 42003_2021_2230_MOESM1_ESM.pdf]

Convergent NMDA receptor – Pannexin1 signaling pathways regulate the interaction of CaMKII with Connexin-36

Ryan C.F. Siu[1,2], Anna Kotova[1,2], Ksenia Timonina[1,2], Christiane Zoidl[1], Georg R. Zoidl[1,2,3*]

Affiliations

1. Department of Biology, York University; Toronto, Ontario, M3J1P3; Canada
2. Center of Vision Research, York University; Toronto, Ontario, M3J1P3; Canada
3. Department of Psychology, York University; Toronto, Ontario, M3J1P3; Canada

***Corresponding author**

Correspondence to gzoidl@yorku.ca

Supplementary Information (available in this document)

Supplementary Methods

Supplementary Figures S1–S8

Supplementary Tables S1–2

Supplementary References

Supplementary Methods:

Plasmid constructs and site-directed mutagenesis **–** Expression vectors with full-length rat Cx36 (NM_019281, amino acids (aa) 1-321), and CaMKIIa (NM_012920.1, aa 1-478) in variations of pEGFP-N1 expression vectors (Clontech Laboratories Inc., Mountain View, CA, USA)[1]. Full-length in-frame cloning of Cx36, the calcium insensitive CaMKIIa mutants: $CaMK_{T286A}$, $CaMK_{T286D}$, $CaMK_{F293A}$, $CaMK_{R296A}$, and the Cx36 mutants: $Cx36_{R278A}$, $Cx36_{K279A}$, $Cx36_{K281A}$, were completed in two steps. The ORFs were synthesized as gBlocks (Integrated DNA Technologies Inc. (IDT), Coralville, IA, USA) and cloned into the TA cloning vector pJet1.2 (Thermo Fisher Inc., Mississauga, ON, Canada). The Cx36 coding regions were then isolated and cloned in-frame into pECFP-N1 or the pDsRed2-monomer expression vectors. We have previously reported the plasmid pEYFP-mPanx1 containing the full-length coding region of the mouse Panx1 (NM_019482)[2]. Here, the EYFP open reading frame was replaced by the far-red fluorescent protein E2-Crimson[3]. All Cx36 and CaMKIIa mutants were generated with the Q5™ Site-Directed Mutagenesis Kit based on the manufacturer's protocol (New England Biolabs Inc. (NEB), Boston, MA, USA). Oligonucleotides were designed with the NEBaseChanger tool (NEB) and synthesized by IDT. **Supplementary Table S2** summarizes the oligonucleotides utilized in this study (mutations depicted in bold and underlined). All plasmid constructs used in this study were sequence verified (Eurofins MWG Operon LLC, Huntsville, AL, USA).

Confocal Microscopy **–** The 48h post transfected cells were fixed with 4% paraformaldehyde for 20 min at room temperature before washing with 1xPBS and mounted for imaging with ProLong® Antifade mounting media (Thermo Fisher Inc., Mississauga, ON, Canada). The fixed samples were imaged using a Zeiss LSM 700 confocal microscope with a Plan-Apochromat 63x/NA 1.4 Oil DIC M27 objective using the ZEN 2010 program interface. Images were collected by line averaging (4x) at 2048x2048 pixel resolution. The gap junction frequency was quantified as described previously [4], by determining the number of fluorescent-expressing cell pairs against the number of cell pairs expressing GJPs. Images were selected at random with non-overlapping visual fields collected from experimental replicates ($n \geq 3$). The GJ area was quantified using the ImageJ software using the freehand tool to draw the contours of GJs between cell pairs and the measure tool for quantification. Images were combined for presentation using Adobe Photoshop 2020.

RNA Extraction and RT-qPCR **-** Total RNAs were extracted from Neuro2a cells using RNeasy Plus Mini Kit (Qiagen). The iScript Reverse Transcription Supermix (Bio-Rad Laboratories, Mississauga, Canada) was used to reverse transcribe 1μg of total RNA. The cDNA equivalent of 15ng total RNA was analyzed in triplicate by quantitative Real Time-PCR using the SsoAdvanced SybrGreen PCR mix (Bio-Rad). All experiments included a melt curve analysis of PCR amplicons generated in each reaction. Raw cycle threshold values (Ct-values) were exported from the CFX Manager Software (Bio-Rad, Canada), and the relative gene expression was calculated using the Relative Expression Software Tool (REST-2009) [5]. Gene information and primer sequences are listed in **Supplementary Table S1.**

Ethidium Bromide Dye-Uptake Assay **-** Neuro2a cells and KD Panx1 cells were cultured for 48 h in 3.5 cm MatTek glass bottom cell culture dishes. Cells were preincubated in $DMEM^{(-)phenol\ red}$ for 30 minutes prior to imaging. Cells were stimulated with $DMEM^{(-)phenol\ red}$ containing 140mM potassium gluconate (KGlu) for 5 minutes. After the KGlu: $DMEM^{(-)phenol\ red}$ stimulus was

removed, MatTek dishes in pre-warmed fresh medium were placed in a live-cell imaging chamber at 37°C and with 5% CO_2. Ethidium bromide was added to a final concentration of 10μM and dye uptake was measured in a randomly selected field of view for 20 minutes at 1-minute intervals using a Zeiss 700 confocal microscope. The percent increase of dye was calculated using the following equation 1:

$$\text{Fluorescence Increase (\%)} = (\Delta F/F)_{treatment} - \text{Avg}(\Delta F/F)_{baseline}$$

where ΔF is the change in fluorescence after 20 minutes and F is the initial fluorescence intensity.

Supplementary Figures:

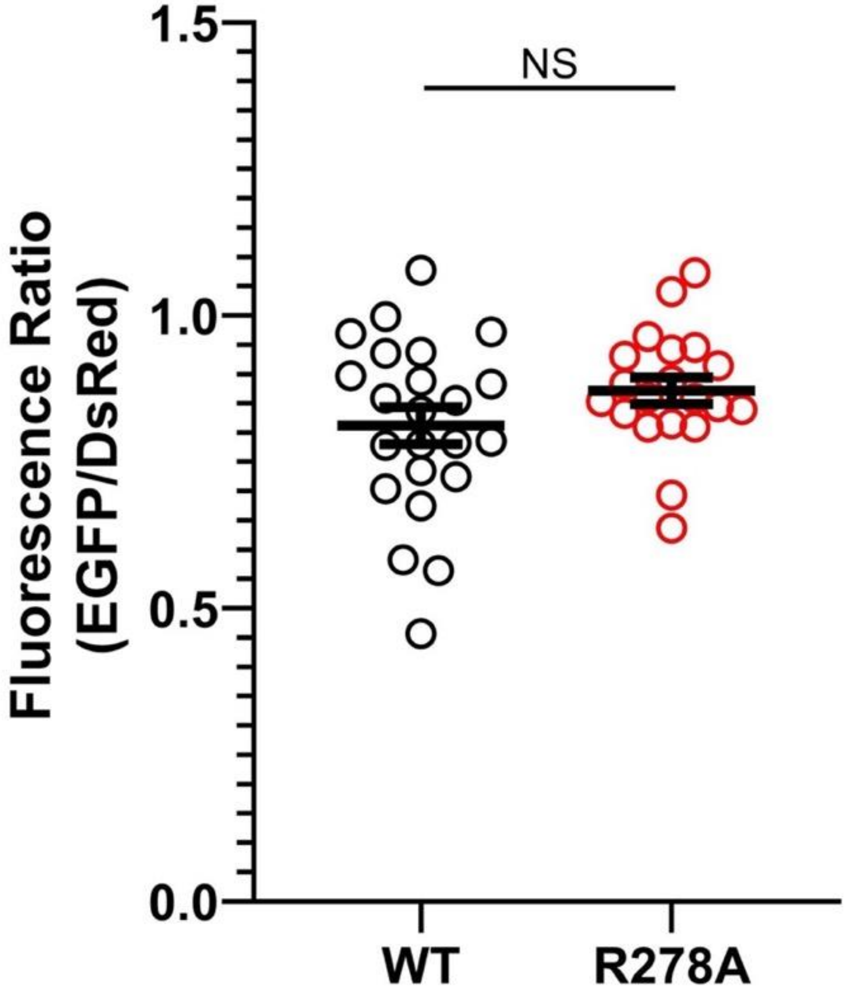

Supplementary Fig. S1: Ratio of EGFP and DsRed fluorescence at GJPs. The fluorescence ratios of Neuro2a cells expressing EGFP or DsRed tagged Cx36 wild type (WT) and R278A proteins were not significantly (NS) different when >20 GJPs were tested. The EGFP/DsRed fluorescence was quantified in ROIs at GJPs. A free-hand line tool in ImageJ was used to overlay GJPs with ROIs. Statistics: Unpaired Students t-test (2-tailed), p=0.14, NS, not significant. Error bars: mean±SEM.

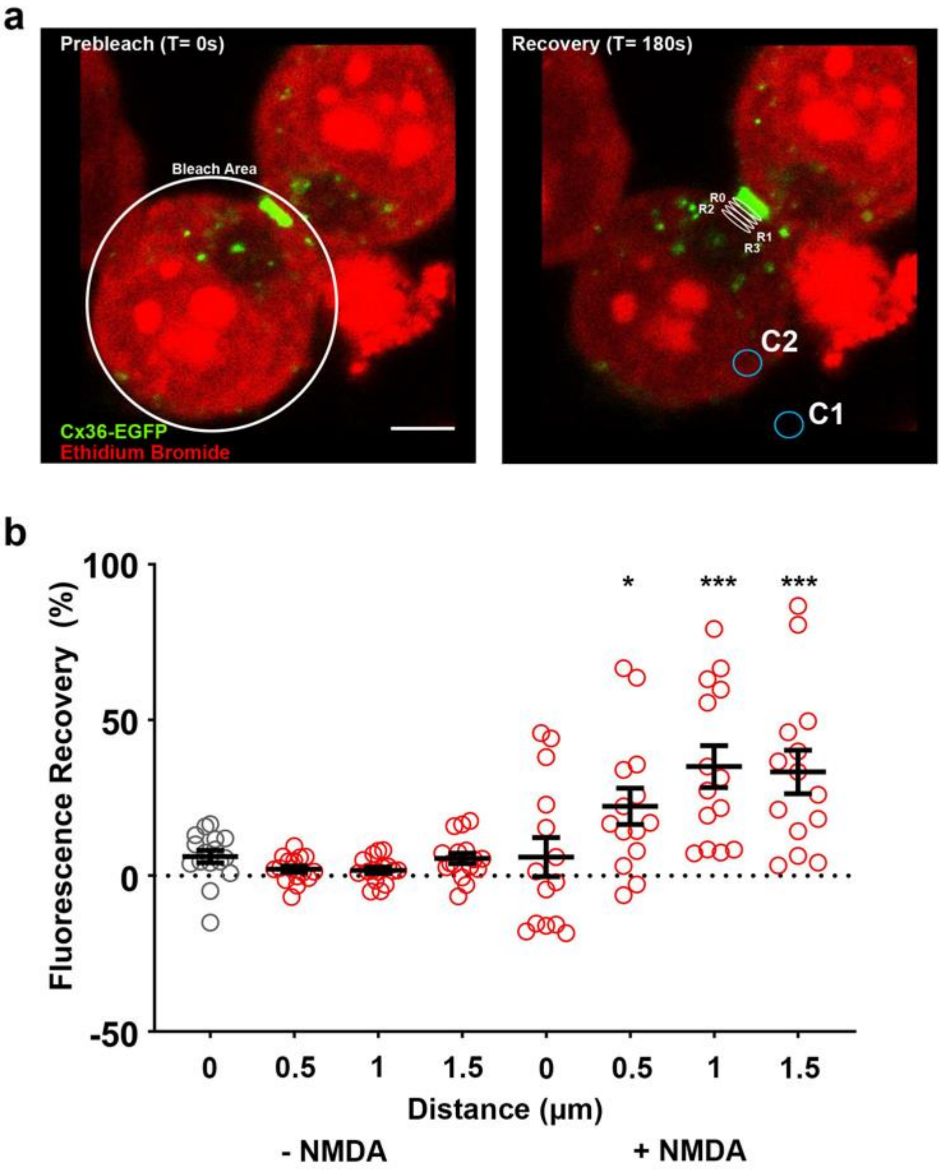

Supplementary Fig. S2: Example of an ethidium bromide uptake and recovery after photobleaching assay showing regions of analysis and the impact of distance on measurements. **a)** On the left, the bleach area in a pair of Cx36-EGFP expressing Neuro2a cells at T=0s is indicated (white ROI). On the right, the same cell is shown after photobleaching and recovery for 180sec. ROIs: R0-3 were placed at 0 – 1.5mm distance to the GJP in the bleached cell. ROI C1 was used for background control. ROI C2 was placed near to the edge of the cell but distant to the GJP (details of quantification see Materials and Methods). **b**) Quantification of fluorescent recovery in the absence (-) or presence (+) of NMDA. n = 14 cell pairs; error bars: mean±SEM. Mann-Whitney U significance (2-tailed), ***$p < 0.001$, *$p < 0.05$.

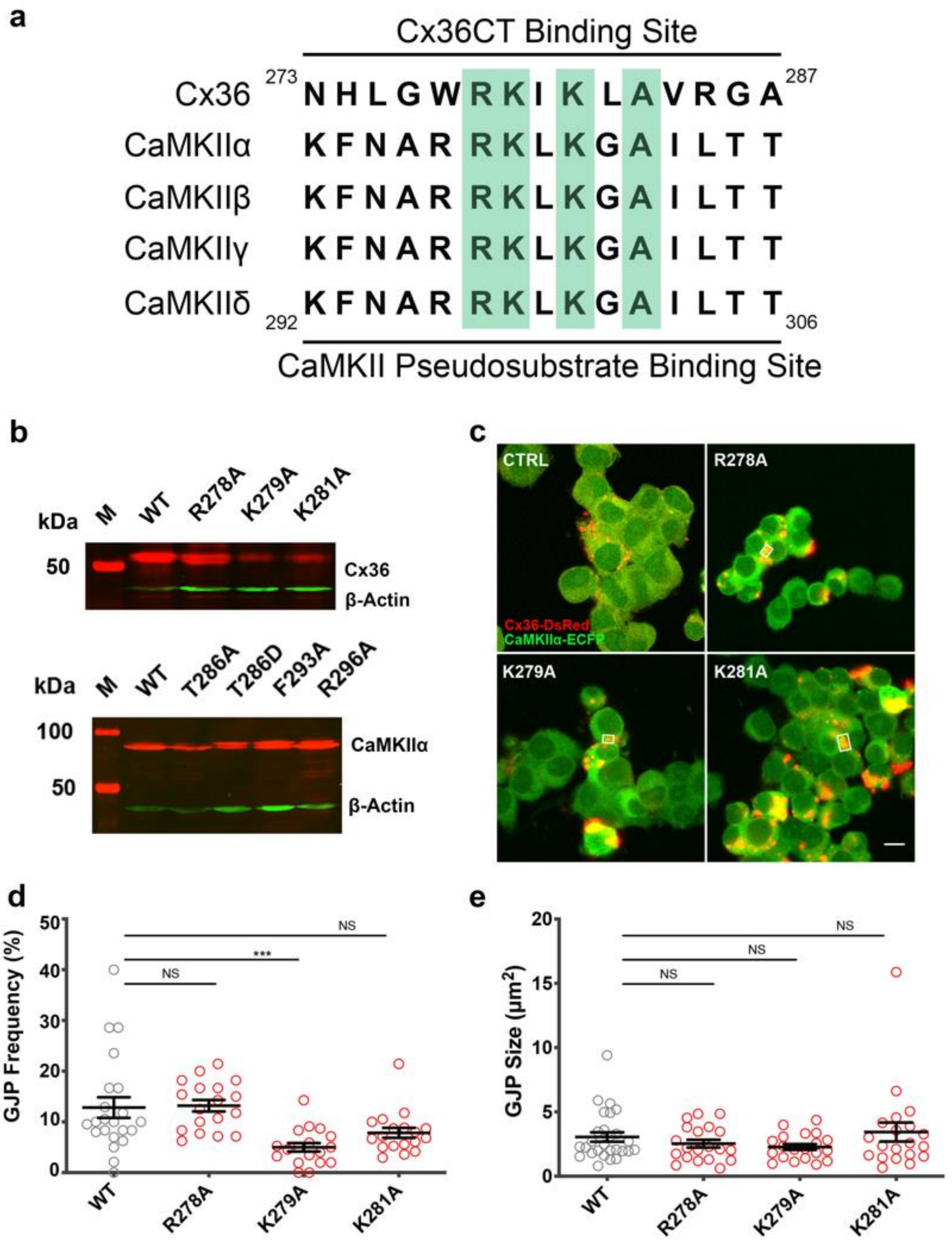

Supplementary Fig. S3: CaMKII and Cx36 mutants showed normal expression in Neuro2a cells. **a)** Alignment of the core region of the pseudosubstrate binding region sequence of CaMKII isoforms (291 to 311) with the Cx36 CT (272 to 292). Conserved amino acids are indicated in green. **b)** Western blot analysis of double transfected Neuro2a cells with CaMKIIα WT, CaMKII mutants (T286A, T286D, F293A, and R296A), Cx36 WT, and Cx36 mutants (R278A, K279A, and K281A). (Red channel = CaMKIIα-EGFP or Cx36-EGFP; green channel = β-actin control) **c)** Images of Neuro2a cells double transfected as outlined in b. Scale bar = 10μm. **(d,e)** GJP frequency and size for Cx36 WT and the Cx36 mutants. Beeswarm graphs with mean±SEM; Mann-Whitney U significance (2-tailed), ***$p < 0.001$, NS, not significant.

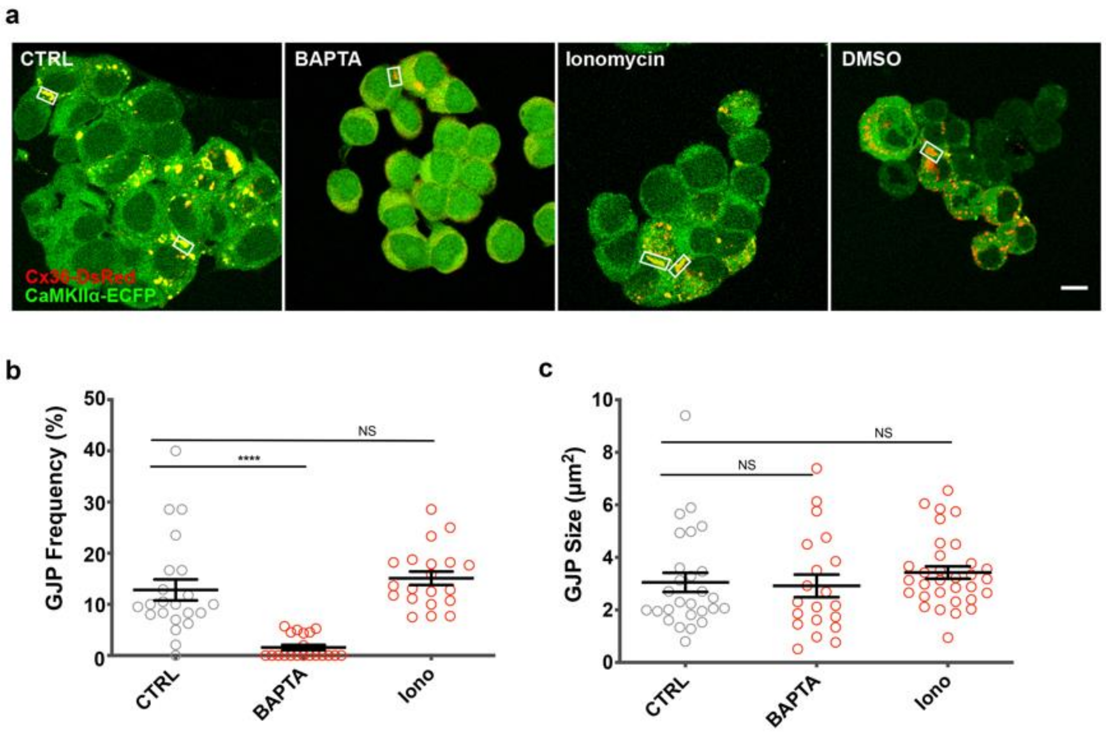

Supplementary Fig. S4: Chelation of calcium reduced GJP frequency but not GJP size of Neuro2a cells expressing CaMKIIα-ECFP and Cx36-DsRed. a) Double transfected Neuro2a cells after treatment with BAPTA-AM, ionomycin, and DMSO. GJPs indicated with a white box. Scale bar = 10µm. **b & c)** GJP frequency and GJP size for treatments outlined above. Beeswarm graphs with mean±SEM; Mann-Whitney U significance, ****$p < 0.0001$, NS, not significant.

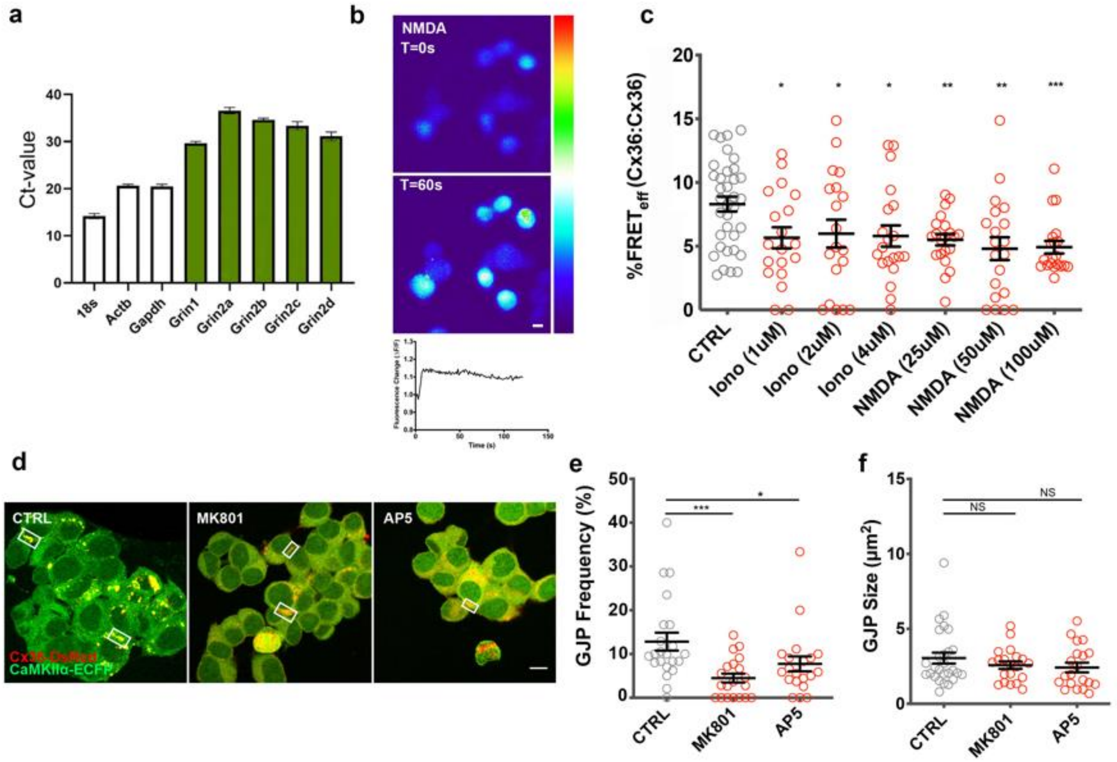

Supplementary Fig. S5: Subunits of NMDA receptors are expressed in Neuro2a cells and respond to stimulants and pharmacological blockers. a) mRNA levels of different ionotropic NMDA receptor subunits in Neuro2a cells. **b)** Rapid influx of calcium (OGB) following NMDA stimulation of NMDA receptors. Sample trace represents relative fluorescence (ΔF/F) of $[Ca^{2+}]_I$ in ROI. Scale bar = 10μm **c)** Dose-dependent decrease in $FRET_{eff}$ contrasting non-selective (ionomycin) and selective (NMDA) increase of $[Ca^{2+}]_I$. **d)** Images of double transfected Neuro2a cells with and without NMDA receptor antagonist treatments. GJPs were highlighted in rectangles. Scale bar = 10μm. Quantification of **e)** GJP frequency and **f)** GJP size of double transfected Neuro2a cells expressing CaMKIIα-ECFP and Cx36-DsRed post-treatment with NMDA receptor blockers. Beeswarm graphs in **b,e,f):** mean±SEM; Mann-Whitney U significance, ***p < 0.001, **p < 0.01, *p < 0.05, NS, not significant.

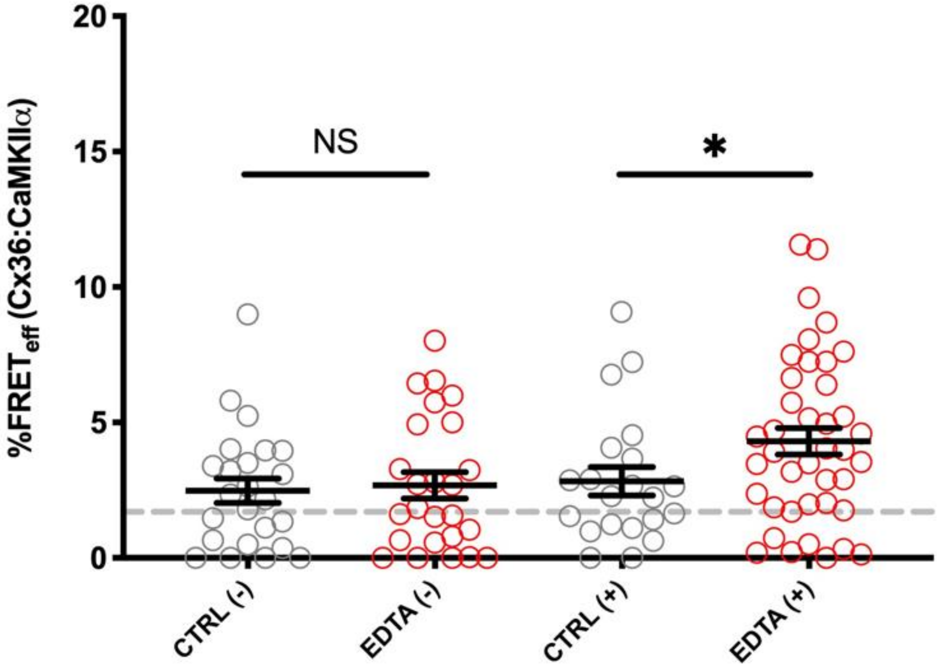

Supplementary Fig. S6: FRET$_{eff}$ of the Cx36-CaMKIIα interaction complex in the presence (+) and absence (-) of non-essential amino acids. Neuro2a cells were co-transfected with Cx36-DsRed and CaMKIIα-ECFP. The FRET efficiency (FRET$_{eff}$) was determined at 48hrs post-transfection. FRET$_{eff}$ was measured without (CTRL) or after treatment with EDTA (3mM) in the presence of standard growth medium supplemented with non-essential amino acids (+) or in medium without (-). In all conditions, the cell culture medium was replaced 30 min prior to the FRET experiment. An increase in FRET$_{eff}$ after the addition of EDTA can be attributed to the cocktail of non-essential amino acids which include glutamic acid and glycine (CTRL (+): 2.835±0.5224, n=21; EDTA (+): 4.302±0.4873, n=40, p=0.0466). When medium was used without non-essential amino acids, EDTA had no effect on the FRET$_{eff}$ (CTRL (-): 2.484±0.4500, n=24; EDTA (-): 2.684±0.4901, n=25, p=0.8620). Beeswarm graph with mean±SEM; dashed line indicates the 1.7% limit of FRET; Mann-Whitney U significance (2-tailed), *p < 0.05, NS, not significant.

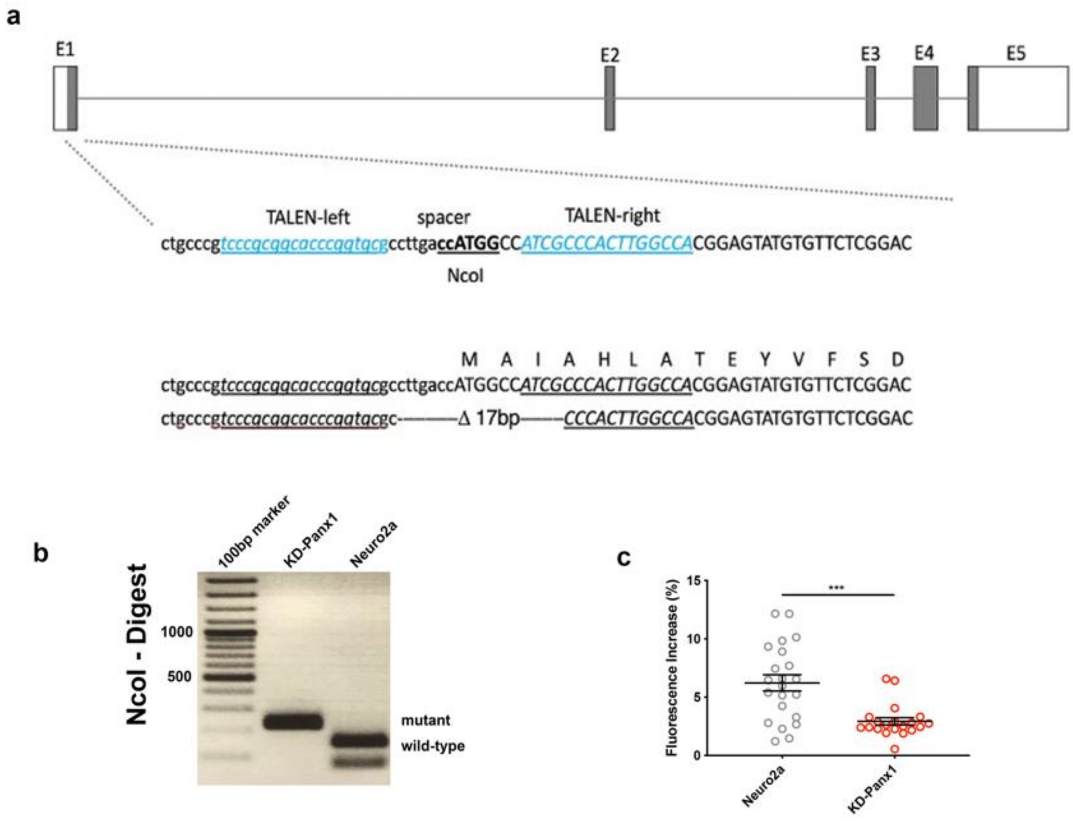

Supplementary Fig. S7: Generation of a Neuro2a cell line with knock-down of Panx1. a) The genome locus of the mouse Panx1 gene (ENSMUSG00000031934). A single NcoI restriction endonuclease recognition site in exon 1 (E1) overlaps with the start codon of the Panx1 protein. This site was selected for gene-edited using a TALEN pair. The position of the left and right TALEN recognition site and spacer are indicated. After gene-editing, a Neuro2a cell line with a 17bp deletion was selected for further experiments. The deletion effectively eliminated the start codon of the Panx1 protein. **b)** Diagnostic restriction enzyme (NcoI) digest of the 226bp DNA-PCR amplicon overlapping the gene-edited exon1 from Neuro2a cells and KD-Panx1 cells confirmed the deletion of the NcoI restriction endonuclease recognition site in gene-edited cells. **c)** Dye uptake assay with Neuro2a and KD-Panx1 cells with/without treatment (5 minutes 140mM KGlu) over 20 minutes using 10µM ethidium bromide. The percent increase of treated cells adjusted to baseline showed that Neuro2a cells uptake more ethidium bromide than KD-Panx1 cells when activated. ***$p < 0.001$.

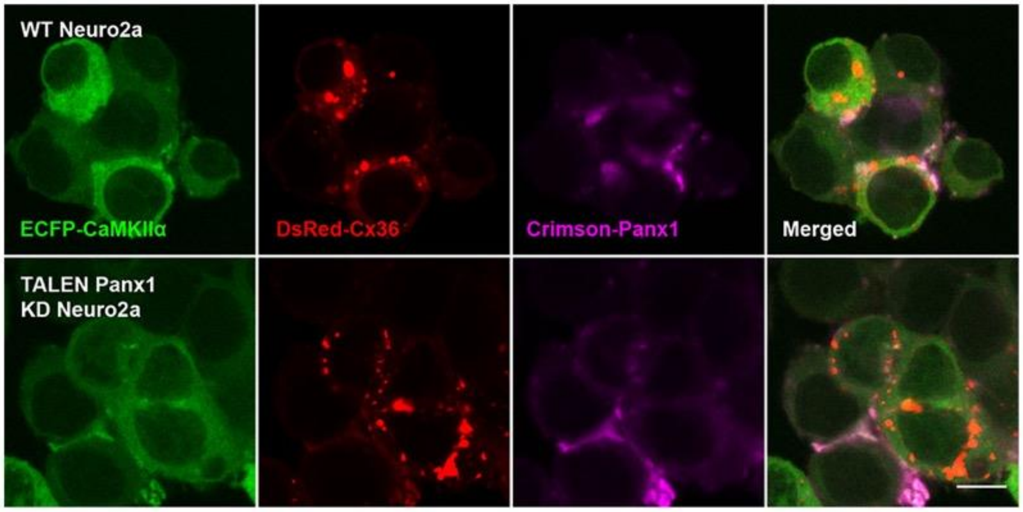

Supplementary Fig. S8: ***Protein localization after triple transfection.*** Representative localization of tagged Cx36-DsRed monomer, CaMKII-ECFP, and Panx1-E-Crimson, after triple transfection into wild-type Neuro2a and KD-Panx1 cells. Scale bar = 10µm.

Supplementary Tables:

***Supplementary Table S1**: Primers for RT-qPCR*

Gene	GeneID	Forward Primer (5'-3')	Reverse Primer (5'-3')
Grin1	NM_008169	TGGGCTTGACATACACGAAG	GCCAGGAGGAGAGACAGAGA
Grin2a	NM_008170	TGGAAGCTCCAAACTGGAAG	CCTCACAGACTTTCATCCCC
Grin2b	NM_008171	GCCAAACTGGAAGAACATGG	TCTGCTCAGACTCTCACCCC
Grin2c	NM_010350	AGAGCAGGCACAGAGAGAGG	GGCGTTTTGTGCTTGGACTA
Grin2d	NM_008172	CCTGCCTTGAGCTGAGTGAG	CCACGGAGAAGAATATCCGA
18s	X00686	GGACTCTTTCGAGGCCCTGTAATTG	TGGAATTACCGCGGCTGCTG
Actb	NM_007393	ATGGAGGGGAATACAGCCC	TTCTTTGCAGCTCCTTCGTT
Gapdh	NM_001289726	CCACTCACGGCAAATTCAAC	CTCCACGACATACTCAGCAC

***Supplementary Table S2**: Oligonucleotides for mutagenesis of CaMKIIa and Cx36*

Gene	Mutation	Forward Primer	Reverse Primer
CaMKIIa	T286A	CAGACAGGAG**GCC**GTGGACTGCC	TGCATGCAGGAGGCCACAG
	T286D	CAGACAGGAG**GAC**GTGGACTGCCTG	TGCATGCAGGAGGCCACA
	F293A	CCTGAAGAAG**GCC**AATGCCAGGAGG	CAGTCCACGGTCTCCTGT
	R296A	GTTCAATGCC**GCG**AGGAAACTGAAGGG	TTCTTCAGGCAGTCCACG
Cx36	R278A	TCTGGGATGG**GCG**AAGATCAAACTGGC	TGGTTAAGTTCAGCCAGATTG
	K279A	GGGATGGCGG**GCG**ATCAAACTGG	AGATGGTTAAGTTCAGCC
	K281A	GCGGAAGATC**GCA**CTGGCTGTCC	CATCCCAGATGGTTAAGTTC

Supplementary References

1 Siu, R. C. et al. Structural and Functional Consequences of Connexin 36 (Cx36) Interaction with Calmodulin. Front Mol Neurosci 9, 120, doi:10.3389/fnmol.2016.00120 (2016).

2 Whyte-Fagundes, P. et al. A Potential Compensatory Role of Panx3 in the VNO of a Panx1 Knock Out Mouse Model. Front Mol Neurosci 11, 135, doi:10.3389/fnmol.2018.00135 (2018).

3 Strack, R. L. et al. A rapidly maturing far-red derivative of DsRed-Express2 for whole-cell labeling. Biochemistry 48, 8279-8281, doi:10.1021/bi900870u (2009).

4 Qu, C., Gardner, P. & Schrijver, I. The role of the cytoskeleton in the formation of gap junctions by Connexin 30. Exp Cell Res 315, 1683-1692, doi:10.1016/j.yexcr.2009.03.001 (2009).

5 Pfaffl, M. W., Horgan, G. W. & Dempfle, L. Relative expression software tool (REST) for group-wise comparison and statistical analysis of relative expression results in real-time PCR. Nucleic Acids Res 30, e36, doi:10.1093/nar/30.9.e36 (2002).
