# Supplementary material for: Convergent NMDA receptor—Pannexin1 signaling pathways regulate the interaction of CaMKII with Connexin-36
Source: Commun Biol. 2021 Jun 8;4:702. doi: 10.1038/s42003-021-02230-x (PMC8187354; doi:10.1038/s42003-021-02230-x)
Supplement: Supplementary file 2 — Description of Supplementary Files [file 42003_2021_2230_MOESM2_ESM.pdf]

Description of Additional Supplementary Files

File name: Supplementary Data 1

Description: Summary of source data for Figs. 1-7, and the Supplementary Figs. S1-S7.
