# Supplementary material for: Convergent NMDA receptor—Pannexin1 signaling pathways regulate the interaction of CaMKII with Connexin-36
Source: Commun Biol. 2021 Jun 8;4:702. doi: 10.1038/s42003-021-02230-x (PMC8187354; doi:10.1038/s42003-021-02230-x)
Supplement: Supplementary file 4 — Reporting Summary [file 42003_2021_2230_MOESM4_ESM.pdf]

nature research

Corresponding author(s): Georg R. Zoidl

Last updated by author(s): Apr 26, 2021

Reporting Summary

Nature Research wishes to improve the reproducibility of the work that we publish. This form provides structure for consistency and transparency in reporting. For further information on Nature Research policies, see our Editorial Policies and the Editorial Policy Checklist.

Statistics

For all statistical analyses, confirm that the following items are present in the figure legend, table legend, main text, or Methods section.

n/a	Confirmed	
☐	☒	The exact sample size (*n*) for each experimental group/condition, given as a discrete number and unit of measurement
☐	☒	A statement on whether measurements were taken from distinct samples or whether the same sample was measured repeatedly
☐	☒	The statistical test(s) used AND whether they are one- or two-sided<br>*Only common tests should be described solely by name; describe more complex techniques in the Methods section.*
☐	☒	A description of all covariates tested
☐	☒	A description of any assumptions or corrections, such as tests of normality and adjustment for multiple comparisons
☐	☒	A full description of the statistical parameters including central tendency (e.g. means) or other basic estimates (e.g. regression coefficient) AND variation (e.g. standard deviation) or associated estimates of uncertainty (e.g. confidence intervals)
☐	☒	For null hypothesis testing, the test statistic (e.g. F, t, r) with confidence intervals, effect sizes, degrees of freedom and P value noted<br>*Give P values as exact values whenever suitable.*
☒	☐	For Bayesian analysis, information on the choice of priors and Markov chain Monte Carlo settings
☒	☐	For hierarchical and complex designs, identification of the appropriate level for tests and full reporting of outcomes
☒	☐	Estimates of effect sizes (e.g. Cohen's *d*, Pearson's *r*), indicating how they were calculated

Our web collection on statistics for biologists contains articles on many of the points above.

Software and code

Policy information about availability of computer code

Data collection	Zen Blue & Zen 2010 (Zeiss) software for Confocal Microscope
Data analysis	ImageJ, GraphPad Prism 8, IBM SPSS Statistics 24, Microsoft Excel

For manuscripts utilizing custom algorithms or software that are central to the research but not yet described in published literature, software must be made available to editors and reviewers. We strongly encourage code deposition in a community repository (e.g. GitHub). See the Nature Research guidelines for submitting code & software for further information.

Data

Policy information about availability of data

All manuscripts must include a data availability statement. This statement should provide the following information, where applicable:

- Accession codes, unique identifiers, or web links for publicly available datasets
- A list of figures that have associated raw data
- A description of any restrictions on data availability

All the data are available in Supplementary Data 1 file

Field-specific reporting

Life sciences study design

All studies must disclose on these points even when the disclosure is negative.

Sample size	Sample sizes were determined based on experimental design from previously published methods and chosen to give reproducible data.
Data exclusions	Outliers were determined by GraphPad and excluded from analysis, but remained on graphs.
Replication	All experiments were replicated.
Randomization	Cells were randomly selected for gap junction plaque size and frequency quantification in multiple batches of cell preparations.
Blinding	Blinding was not applicable for this study since experimenters needed to determine transfected cells prior to any analysis.

Reporting for specific materials, systems and methods

We require information from authors about some types of materials, experimental systems and methods used in many studies. Here, indicate whether each material, system or method listed is relevant to your study. If you are not sure if a list item applies to your research, read the appropriate section before selecting a response.

Materials & experimental systems

n/a	Involved in the study	
☐	☒	Antibodies
☐	☒	Eukaryotic cell lines
☒	☐	Palaeontology and archaeology
☒	☐	Animals and other organisms
☒	☐	Human research participants
☒	☐	Clinical data
☒	☐	Dual use research of concern

Methods

n/a	Involved in the study	
☒	☐	ChIP-seq
☒	☐	Flow cytometry
☒	☐	MRI-based neuroimaging

Antibodies

Antibodies used	Primary antibodies were diluted 1:500 (mouse anti-GFP, Roche; rabbit anti-GFP (FL), Santa Cruz Biotechnologies, TX, USA), 1:1,000 (rabbit anti-Panx1, Invitrogen, 488100) and 1:15,000 (mouse anti-b-actin; Sigma-Aldrich Chemie GmbH, Munich, Germany). The secondary antibodies (LI-COR Biosciences, St. Lincoln, NE, USA) were diluted 1:15,000 (donkey anti-rabbit IRDye680LT) or 1:20,000 (goat anti-mouse IRDye800CW).
Validation	All antibodies are well characterized and commercialized. Antibodies have been extensively used in the lab for many years with consistency.

Eukaryotic cell lines

Policy information about cell lines

Cell line source(s)	Neuro2a
Authentication	*Describe the authentication procedures for each cell line used OR declare that none of the cell lines used were authenticated.*
Mycoplasma contamination	not tested recently (2021)
Commonly misidentified lines (See ICLAC register)	*Name any commonly misidentified cell lines used in the study and provide a rationale for their use.*
